# The Impact of Gel Parameters on the Dispersal and Fragmentation of Hyaluronic Acid Gel Fillers within an Artificial Model of Arterial Embolism

**DOI:** 10.3390/gels10080530

**Published:** 2024-08-12

**Authors:** Danny J. Soares, Alec D. McCarthy

**Affiliations:** 1 College of Medicine, University of Central Florida, Orlando, FL 32827, USA; 2 American Foundation for Aesthetic Medicine, Fruitland Park, FL 34731, USA; 3 Merz Aesthetics Inc., Raleigh, NC 27615, USA; alec.mccarthy@merz.com

**Keywords:** vascular occlusion, arterial embolism, dermal filler injuries, soft tissue ischemia

## Abstract

Accidental arterial embolization of hyaluronic acid (HA) fillers can lead to severe complications, including skin ischemia, blindness, and stroke. Currently, the intra-arterial dispersal and fragmentation behavior of HA gels is unknown but critical to our understanding of the pathomechanism of these injuries. This work introduces the Pulsatile Unit for the Laboratory Simulation of Arterio-embolic Restrictions (PULSAR) and evaluates the intravascular behavior of different HA gels. The fragmentation and dispersal behaviors of four HA gels with distinct rheological properties were evaluated via high-resolution videography and ImageJ particle size and morphology analysis. The gels’ elastic modulus (G′), loss modulus (G″), tan(δ), and HA concentration were subsequently correlated with their intra-arterial behaviors. This study effectively confirms the extensive fragmentation of HA gels upon arterial inoculation, with particle sizes ranging from <50 µm to >1 mm. Gel particle size and morphology correlated most significantly with tan(δ). Conversely, arterial flow rates did not significantly influence gel fragmentation behavior, though the probability of proximal, macrovascular obstruction was affected. Overall, this study validates the PULSAR model for simulation of arterial dynamics and the testing of intravascular filler kinematics. The findings demonstrate the ability of gels to microfragment and disseminate distally, as well as induce partial proximal occlusion depending on gel rheology and arterial flow parameters.

## 1. Introduction

**Figure 1:**
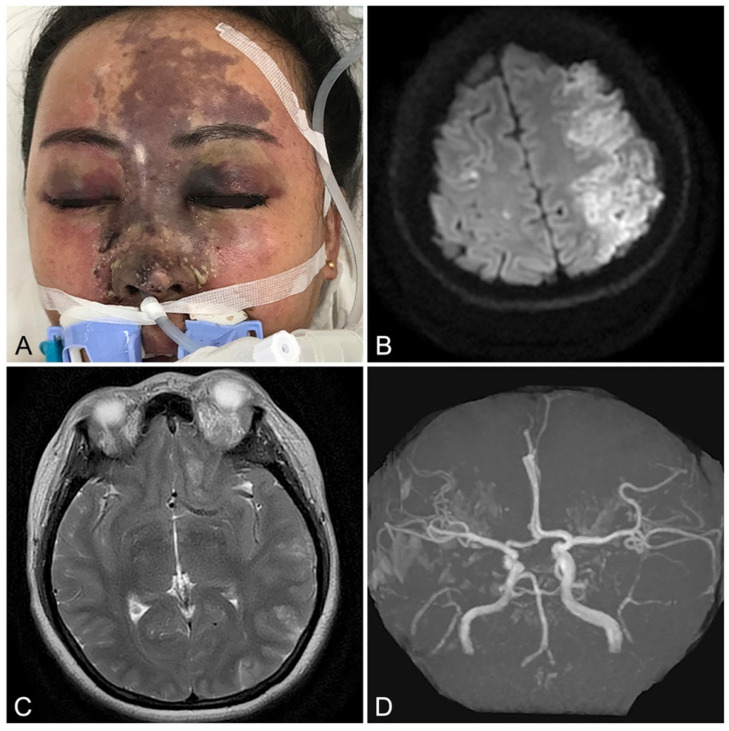
Example of extensive facial soft tissue ischemia with neuro-ophthalmological sequelae resulting from iatrogenic filler embolization: (**A**) Appearance of 40-year-old female 5 days following arterio-embolic injury resulting from dorsal nasal hyaluronic acid filler augmentation, revealing ischemic changes of the frontonasal skin of the face. (**B**) Diffusion-weighted axial magnetic resonance imaging (MRI) demonstrating left hemi-frontoparietal cerebral infarction. (**C**) T2-weighted axial MRI revealing edema and ischemic changes affecting the left optic nerve and orbital soft tissues. (**D**) Magnetic resonance angiography demonstrating intact proximal cerebral circulation (circle of Willis and anterior, middle, and posterior cerebral arteries), suggesting a distal embolic etiology. Reproduced with permission from Yang et al. [3], 2020, Springer Nature.

Iatrogenic arterial embolization stemming from accidental intravascular injection of therapeutic colloids and suspensions represents a potentially devastating medical etiology leading to acute soft tissue infarction [1]. Depending on the affected vessel, the resultant negative sequelae can range from limb loss and facial disfigurement (known as *embolia cutis medicamentosa* or *Nicolau syndrome*) to pulmonary embolism, blindness, and acute cerebral stroke (Figure 1) [2,3,4,5]. Despite its rare incidence, the frequency of these injuries has risen substantially owing to the recent growth in the popularity of injectable gel implants, specifically hyaluronic acid (HA) colloidal mixtures employed in cosmetic facial rejuvenation and orthopedic joint viscosupplementation [6,7]. The pathomechanism of injury is presumed to arise from the inadvertent gel inoculation of an artery with subsequent localized occlusion and/or distal dispersal of embolic fragments [8,9,10]. Given the variable nature of arterial networks, which demonstrate differing degrees of vascular redundancy, perfusion rates, and ischemic tolerance, the occurrence and magnitude of tissue injury can range from minor to extensive [11,12,13].

The recent, alarming 30-fold increase in the incidence of this devastating condition has motivated new efforts to elucidate its primary mechanism of injury, highlighted by a dedicated United States Food and Drug Administration (FDA) panel on the subject, with the goal of informing future preventative and therapeutic interventions [6,14]. Etiologically, the rate and degree to which HA gels fragment and disperse upon arterial inoculation represents a potentially critical factor influencing the point of occlusion and mechanism of tissue underperfusion/ischemia, potentially offering insight into treatment strategies [15]. Nonetheless, despite a comprehensive, application-specific body of literature on the physicochemical parameters and *in vivo* behavior of these implantable gels, little is known regarding their behavior as intravascular embolic agents, including their fragmentation, dispersal, and occlusive potential under physiological, human conditions [16,17,18]. In this study, we introduce the Pulsatile Unit for the Laboratory Simulation of Arterio-embolic Restrictions (PULSAR) as an artificial model of facial arterial HA gel inoculation and evaluate the embolic particularization and dispersibility of four HA gels of differing strengths and viscoelastic profiles.

## 2. Results

### 2.1. PULSAR System Calibration

**Figure 2:**
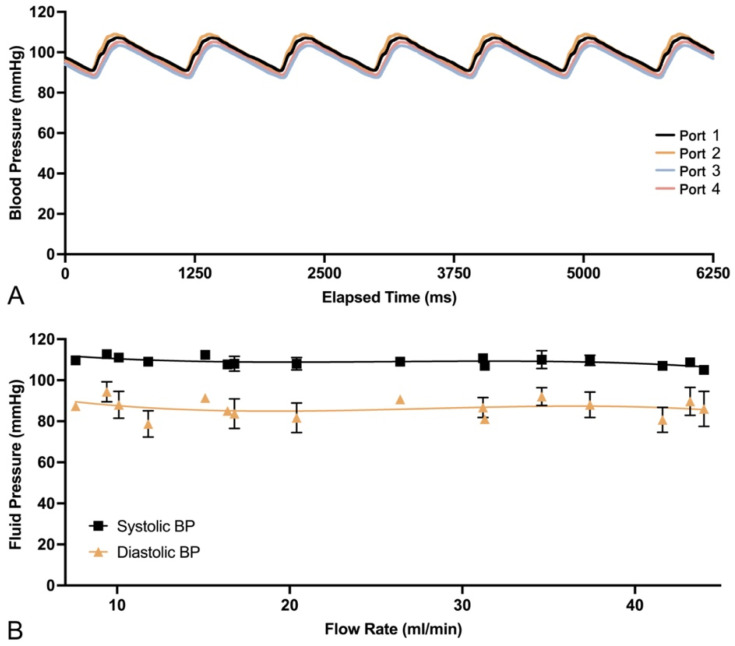
Data plots for PULSAR system calibration: (**A**) Illustrative pressure waveform for all four distal ports (1–4) during system activation at 65 bpm. (**B**) Summary plot of average systolic and diastolic fluid pressures relative to flow rate, showing the ability of the system to maintain physiological pressures at different flow rates.

PULSAR system calibration demonstrated a pulsatile pressure waveform (Figure 2), with an average pulse pressure of 22 ± 5 mmHg, maintained through the target ranges for heart rate and stroke volume (Table S1). Systemic fluid pressures averaged 109/86 mmHg ± 3/6 mmHg (range: 95–114/66–100 mmHg, MAP: 93.5 mmHg) spanning from 110/83 ± 2/3 mmHg at 60 bpm to 107/90 mmHg ± 1/2 mmHg at 100 bpm. Port 1 flow-rate measurements registered between 7.6 and 44 mL/min, remaining largely within physiological flow rates for the paramandibular facial artery, with laminar flow confirmed visually (please see Video S1) and corroborated by an average Reynold’s number of 44 ± 23 across all settings. Flow-specific settings for the facial artery simulation, established for the experimental inoculation, are shown in Table 1, with low, medium, and high flow rates set at 7.4, 17.6, and 35.8 mL/min, respectively.

### 2.2. Gel Particle Size and Morphology Analysis

**Figure 3:**
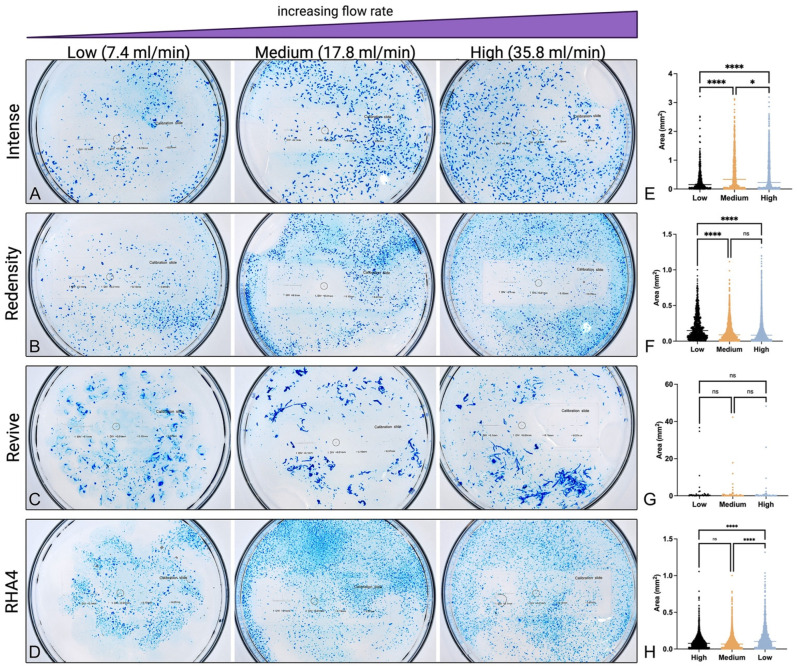
Appearance and size distribution of embolic gel particles for each of the four tested products: (**A**–**D**) High resolution photographic images of embolized gel fragments in returning fluid collected immediately following system inoculation with Belotero Intense, RHA Redensity, Belotero Revive, and RHA4 filler products at low, medium, and high flow rates, respectively. (**E**–**H**) Particle size distributions for each product at different flow rate settings. Significance denoted as *p* < 0.05 (*), and *p* < 0.0001 (****).

**Figure 4:**
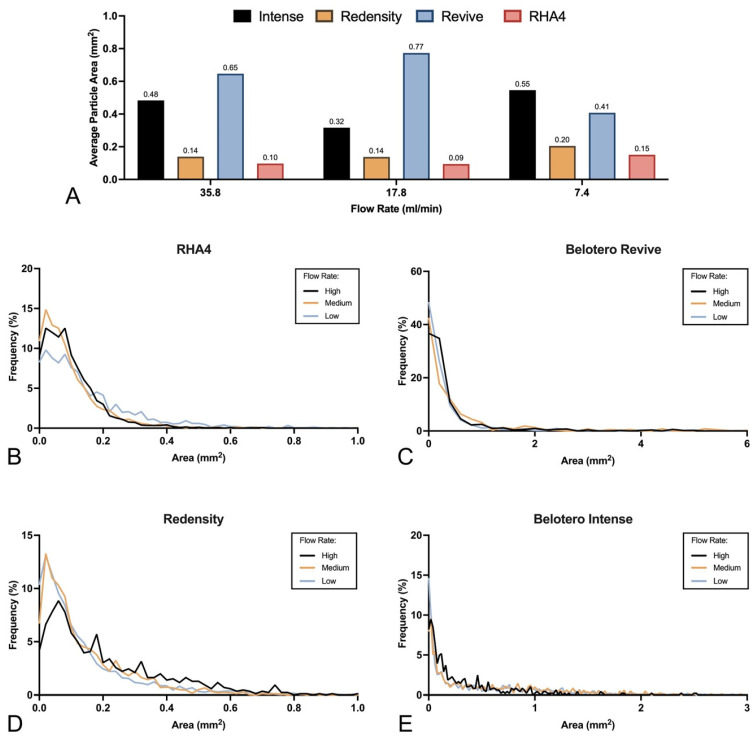
Average size and distributions for all tested fillers at each flow rate: (**A**) Average particle size for each product at low, medium, and high flow rates. (**B**–**E**) Frequency distributions for gel particle sizes at low, medium, and high flow rates for RHA4, Belotero Revive, RHA Redensity, and Belotero Intense, respectively.

Post-inoculation filler fragmentation analysis is visually displayed in Figure 3, with dimensional and shape data summarized in Table 2. All filler products demonstrated extensive fragmentation upon intravascular injection and dispersal at all flow rates, with an average fragment size of 0.316 ± 0.527 mm^2^ (interquartile range (IQR): 0.041–0.378 mm^2^) and average fragment dimensions of 356 ± 294 µm (IQR: 149–497 µm) in minor axis and 596 ± 553 µm (IQR: 225–801 µm) in major axis. Notably, Belotero Revive formed long filamentous fragments interspersed with smaller particles at all flow rates, while all other fillers assumed more uniform microfragment size and shape. Particle size distribution frequencies are presented in Figure 4. Fragments varied significantly in size according to filler brand, with PNT products yielding smaller particles (RHA4: 0.113 ± 0.096 mm^2^; RHA Redensity: 0.160 ± 0.162 mm^2^) compared to CPM gels (Belotero Intense: 0.448 ± 0.525 mm^2^; Belotero Revive: 2.316 ± 7.627 mm^2^, *p* < 0.0001). Additionally, minor flow-rate-dependent variations in particle size were encountered, with PNT products displaying a greater tendency toward microfragmentation with increasing flow rates compared to CPM products, which consistently maintained relatively large particle sizes at all flow rates (Figure 4A). The distribution histograms for each filler at each flow rate are displayed in Figure 4B–E, showing fairly uniform particle size frequencies, indicating that flow rate affected the filler-specific distribution of particle sizes only minimally. Finally, microscopic evaluation of sampled fluid confirmed the presence of dyed microparticles ranging from 1 to 50 µm (Figure S1). Pairwise comparisons denoting significance are given in Tables S2–S5.

**Figure 5:**
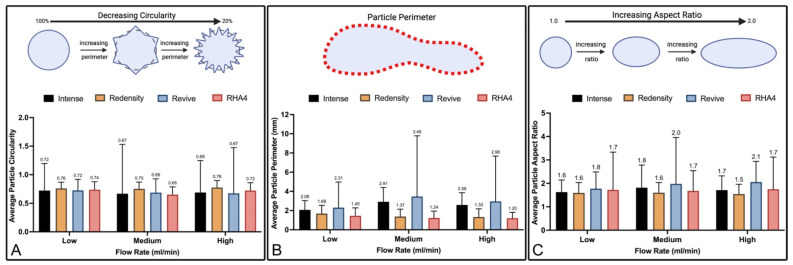
Gel particle morphological analysis: (**A**–**C**) Summary plots of average particle circularity, perimeter, and aspect ratio, respectively, at low, medium, and high flow rates for all products.

Particle morphology analysis data are presented in Figure 5, providing an additional visual representation of gel particle circularity, perimeter, and aspect ratios already summarized in Table 2. Circularity is a measure of how close a particle is to a perfect circle, with a value of 1.0 denoting perfect circularity (Figure 5A). Flow rate had minimal impact on each filler fragment circularity, though filler circularities varied between brands (*p* < 0.0001). Most filler fragments ranged from approximately 0.65 to 0.75, regardless of brand and flow rate, indicating moderate circularity, with PNT products showing higher values compared to CPM (0.73 vs. 0.69, respectively). Similarly, the perimeter of each gel’s fragments was minimally impacted by flow rate but varied significantly between filler products (Figure 5B). Belotero Intense ranged from 2.08 to 2.91 mm over low, medium, and high flow rates, while Redensity, Revive, and RHA4 ranged from 1.327 to 1.682 mm, 2.306 to 3.461 mm, and 1.202 to 1.454 mm, respectively. At all flow rates, Revive particles demonstrated the largest perimeters, given their more filamentous shape, followed by Intense, Redensity, and RHA4. Similar findings were also encountered with particle aspect ratios (Figure 5C), with Belotero Revive having the highest average aspect ratio, which correlated with the visible strand-like gel fragments observed under magnification. Across all flow rates, Redensity had the lowest average aspect ratio; however, the majority of gel fragments had aspect ratios between 1.5 and 2.1, indicating that the average fragment for all gel products at all flow rates is ovoid rather than circular. A summary of pairwise comparisons for size and morphology analysis can be found in their respective Tables S6–S8.

### 2.3. Correlates with Fragmentation Behavior

**Figure 6:**
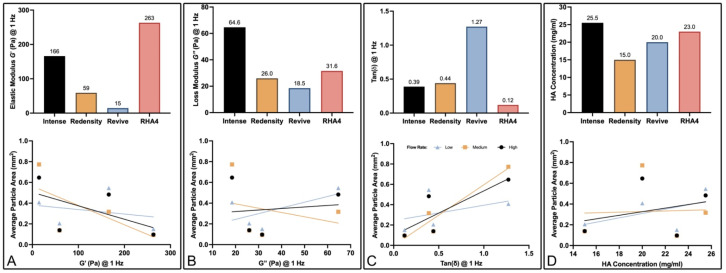
HA gel physical and rheological properties and their correlations with average particle size at low, medium, and high flow rates: (**A**) Elastic modulus. (**B**) Loss modulus. (**C**) Tan(ẟ). (**D**) HA concentration and their correlation plots.

**Figure 7:**
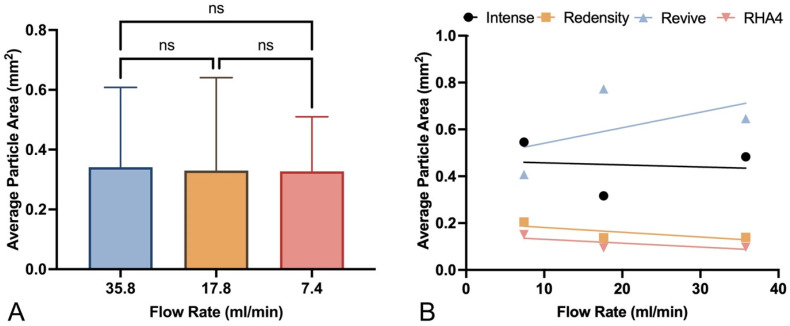
Evaluation of the effect of flow rate on average HA gel particle fragment size: (**A**) Average particle size for all HA gel products at low, medium, and high flow rates (ns—not statistically significant). (**B**) Correlation of flow rates and average particle size for individual HA gel products.

**Figure 8:**
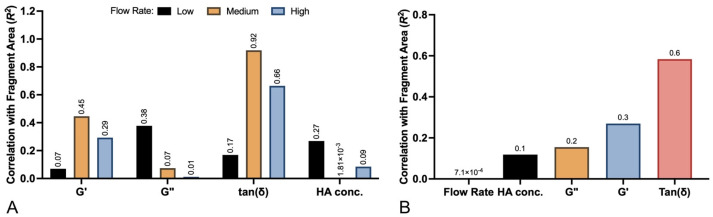
Comparison of correlation coefficients between gel physical properties and flow rates on particle size: (**A**) Correlation coefficients between G′, G″, tan(ẟ), and HA concentration and average particle size at each flow rate. (**B**) Overall correlation coefficients with average particle size for all combined rates.

To elucidate the primary gel parameters affecting the fragmentation behavior of different filler products, a variety of regression analyses were conducted to evaluate potential correlation with key physical properties, including elastic modulus (G′), loss modulus (G″), tan(δ), and HA concentration, displayed in Figure 6A–D. Regression analyses between gel fragment size and filler properties were also carried out for each flow setting. The tan(δ) parameter demonstrated the highest correlation with fragment size (maximum R^2^ = 0.92 at medium flow), followed by G′ (maximum R^2^ = 0.45 at medium flow), G″ (maximum R^2^ = 0.38 at medium flow), and HA concentration (maximum R^2^ = 0.27 at high flow). Summary data for each regression analysis are listed in Table 3. To determine the role of flow rate on fragment size, an additional regression analysis was performed, this time combining the fragments from all fillers. The average particle size for all fillers at low, medium, and high flow rates did not significantly differ (Figure 7A). When isolating each filler product, flow rate still did not significantly correlate with fragmentation size. The best fit between fragment size and flow rate was 0.577, which occurred with Redensity (Figure 7B). The correlation between all filler fragments and flow rate was much poorer, with an R^2^ value of 0.001. Summarized average R^2^ values at each flow rate and for each physical property are displayed in Figure 8A. Thus, when averaging across all flow rates, R^2^ values, in increasing order, were 0.001, 0.119, 0.156, 0.270, and 0.584 for flow rate, HA concentration, G″, G′ and tan(δ), respectively (Figure 8B).

### 2.4. Dynamic Evaluation of Occlusive Potential of Filler Products

**Figure 9:**
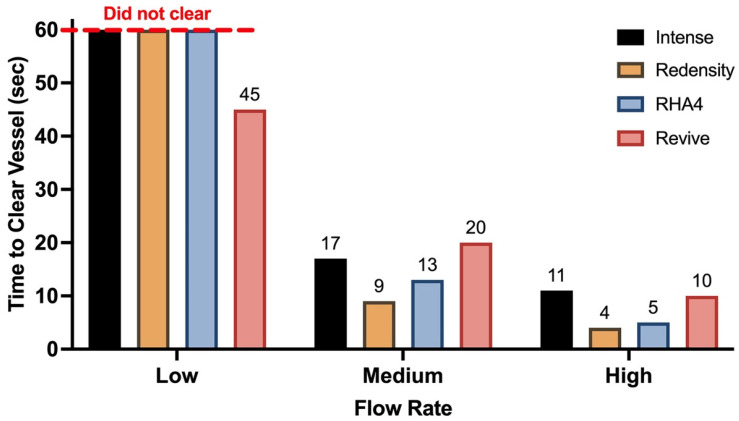
Clearance time of embolic burden for each product at different flow rates.

**Figure 10:**
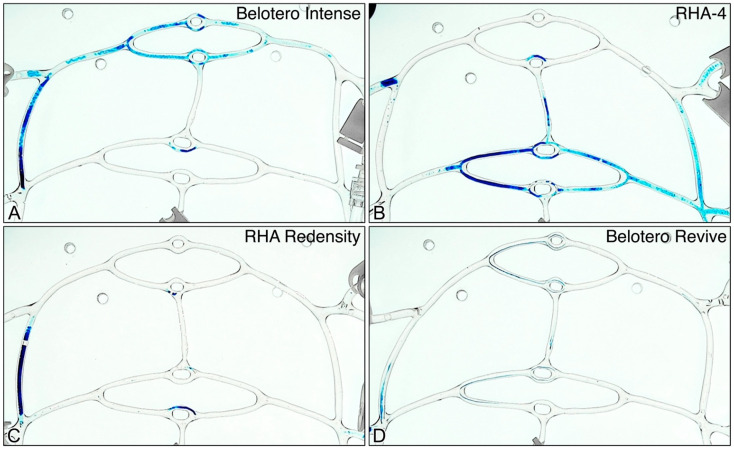
Appearance of obstructive gel plugs and non-obstructive residue following inoculation for all products at low flow rate (7.4 mL/min): (**A**–**D**) Obstructive gel plugs for Belotero Intense, RHA4, RHA Redensity, and Belotero Revive, respectively, showing obstructive branch occlusion for Belotero Intense, RHA4, and Redensity and only non-obstructive residue for Belotero Revive.

Videographic data for all gel product inoculations are summarized in Videos S2–S5, with circuit clearance time plotted in Figure 9. All gels easily cleared the circuit fully at medium and high flow rates, though Revive, and to a lesser degree Redensity, left visible non-obstructive residue on the tubing walls. At low flow rates, RH4, Redensity, and Intense resulted in complete blockage of one of the first-generation daughter branches, though unimpeded flow through the circuit still continued via non-obstructed branches (Figure 10). In contrast, Belotero Revive did not cause permanent obstruction of any branches at the low flow rate, clearing the circuit fully, with only non-obstructive residue left behind.

## 3. Discussion

**Figure 11:**
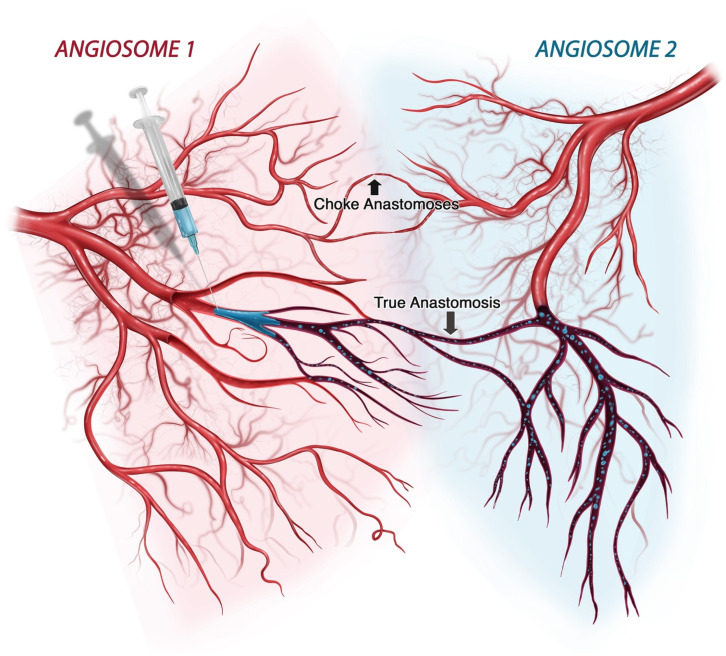
Diagrammatic representation of vascular redundancy in tissues resulting from anastomotic connections between and within angiosomes. In human soft tissues, particularly the skin and skeletal muscle, the presence of anastomoses can provide adequate perfusion pressure to affected regions and offer bypass routes that circumvent embolic restrictions. Generally, redundancy is afforded through the presence of large-caliber true anastomoses (also known as direct anastomoses) and small-caliber, functional choke anastomoses (also known as indirect anastomoses). Direct anastomoses, such as those that exist between the ophthalmic and facial angiosomes, can also provide a direct route for embolic dissemination of gel particles, potentially enabling the occurrence of retinal ischemic injuries arising from injections in the lower face. Reproduced with permission from Soares [1] 2022, MDPI.

The susceptibility of soft tissues to iatrogenic arterial embolization depends on the degree of tissue tolerance to hypoxemia and the extent of perfusion deficit generated by the embolic load. In humans, arterial networks demonstrate significant regional differences in configuration, reflecting variability in perfusion redundancy [19]. While some tissues exhibit an end-arterial arrangement sustained by one feeding vessel (e.g., retinal and distal cerebral circulation) others feature a robust perfusion system nourished by multiple neighboring angiosomes interconnected via true direct anastomoses (e.g., skin and muscle) (Figure 11) [20,21,22]. Such redundancy, necessary for thermoregulation and functional muscular hyperemia, also plays a protective role in instances of embolic occlusions, demanding a more distal target of blockade and/or larger volumes of gel inocula to sufficiently disrupt microvascular tissue perfusion to the point of ischemia [15]. Conversely, an end-arterial design is potentially vulnerable even to proximal, small-volume occlusions, though even highly-redundant networks become increasingly end-arterial distally. Resultantly, the macrovascular fragmentation profile of an intra-arterial gel bolus, and its potential for microvascular dissemination, play a pivotal role on the spatial distribution of an embolic agent and the likelihood of ischemic injury vs. non-injurious hypoperfusion. Therapeutically, the configuration of an arterioembolic occlusion bears special significance, particularly in instances involving enzymatically degradable HA gels, for which early intervention with hyaluronidase can be curative [23,24]. Thus, knowledge of the fragmentation profile of a gel embolus is necessary to better predict the locus of occlusion and the likelihood of ischemia at any given inoculation volume.

To date, the experimental characterization of iatrogenic arterio-embolic injuries resulting from implantable hydrogels has mostly relied on research within animal models, specifically the rabbit auricular/femoral and rat femoral/epigastric arterial systems, employing live arterial inoculations with a limited number of filler products [25,26,27,28,29,30,31]. Despite clinical and histological evidence suggesting microfragmentation of HA gel inocula with distal dispersal, the extent to which the initial gel bolus is mechanically disintegrated and its rate of dispersion, particle size distribution, and configuration of proximal versus distal occlusion, have not been conclusively established for gels of different mechanical parameters in humans [32,33,34,35,36]. This gap in knowledge is a consequence of the intrinsic limitations within animal model systems, in which high-quality, real-time visualization of the gel behavior is not permissible, non-human physiological parameters are employed, and the testing of multiple gels with differing rheological parameters poses too high a financial, logistical, and animal-sacrifice burden.

In the present study, the establishment of a novel artificial testing environment for the mechanical testing of gel emboli within a physiologically relevant, pulsatile macrofluidic system offers the opportunity for real-time evaluation of gel fragmentation and dispersal. In this study, calibration analysis confirms the ability of the PULSAR system to maintain controlled fluid pressures and specific flow rates within the natural range of human physiology, offering a significant advancement over previous experimental designs [37,38]. Furthermore, given the wide range of vessel diameters encompassed by the PULSAR network, the system can be adapted for studies employing a multitude of vascular parameters. In this study, our inoculation testing design aimed to specifically simulate the paramandibular facial artery and its proximal branches due to the high incidence of injuries (58%) affecting the facial angiosome [6]. Given the variable range of flow rates characteristic of the proximal facial artery, the PULSAR system was also capable of successfully maintaining different flow settings for the evaluation of flow rate effects on the degree fragmentation, particle size, and obstructive potential at low, medium, and high physiological flow rates.

The experimental findings in this study confirm that HA gel fillers can undergo substantial fragmentation into microparticles, with potential for wide dissemination, upon macrovascular inoculation, at all tested flow rates. Embolic particles were largely ovoid, encompassing a large range of sizes, from a few micrometers to greater than 1 mm, with the highest frequency occurring between approximately 60 and 800 μm in minor axis and 90 and 1400 μm in major axis. These findings agree with prior *in vitro* laser diffraction analysis of HA gel particles in aqueous dispersions as well as histological studies suggesting the capacity for arterial inoculations to launch micro-emboli capable of disrupting the distal microcirculation [39,40]. Furthermore, the presence of large particles greater than 1.5 mm was primarily encountered with inoculations employing Belotero Revive, a relatively viscous gel with a high tan(δ) of 1.27 and predominantly fluid behavior, which contrasts with the more elastic gel profile of the other tested products (tan(δ) values of 0.12, 0.39, 0.44). Notably, despite featuring the largest fragment sizes, Belotero Revive did not result in luminal obstruction of any branch within the PULSAR system, regardless of flow rate, consistent with prior rat femoral artery experiments using uncrosslinked HA solutions with tan(δ) > 1 [41].

**Figure 12:**
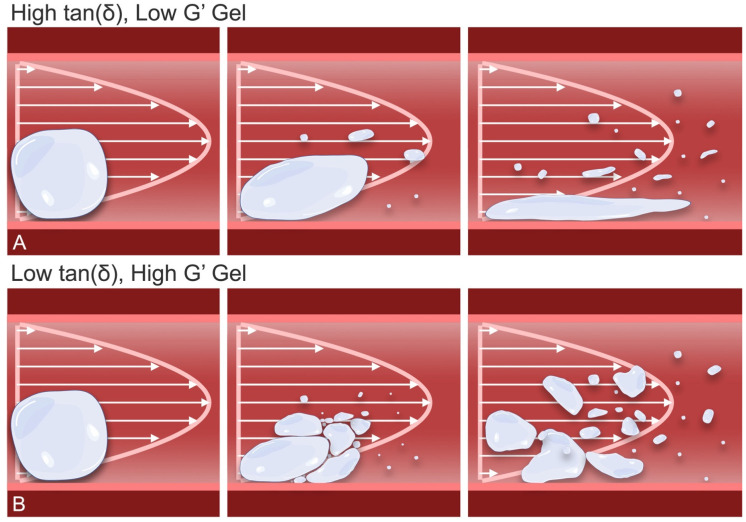
The impact of rheological parameters on the cannulated extrusion and/or intravascular fragmentation of gel particles: (**A**) High-tan(δ), low-G′ gels, having greater pliability, can deform more easily in response to flow-related shear stresses during extrusion through needles/cannulas or dispersal within arterial conduits. The deformation behavior of these gels allows for streamlining of their shape, reducing exposure to shear stress and resulting in long filamentous fragments that fail/break in a ductile manner. (**B**) Low-tan(δ), higher-G′ gels, though stronger and better able to resist deformation and shear stresses, upon exposure to the sufficiently high shear stresses of needle extrusion or intra-arterial environments, are unable to deform to the same extent as softer, more pliable gels. As a result, these gels tend to fail in a more brittle manner, progressively fragmenting into smaller, non-filamentous particles.

The likely cause for the reduced macro-occlusive capacity of relatively viscous, fluid HA products like Belotero Revive relates to their ability to easily deform with flow [16,42]. The combination of low gel strength and high viscous partitioning renders the product highly pliable, allowing the gel to adaptively streamline its shape in response to intraluminal stress differentials, reducing its exposure to shear stress and deforming into long filamentous strands without achieving permanent macrovascular occlusion (Figure 12A). Nonetheless, continuous distal dispersal of these thin gels is likely to result in eventual microfragmentation through brittle or extensional failure, suggesting that they may ultimately disperse into even smaller microparticles (<100 μm) within human microvascular networks, as previously histologically documented [43]. Additional studies employing microfluidic networks will offer further insight into the dynamic microvascular behavior of gels like Revive. Importantly, despite their greater ability to evade proximal flow obstruction, thin uncrosslinked HA gels may still result in distal microvascular occlusion at sufficiently small vessel diameters, though their low degrees of chemical crosslinking are also likely to permit an accelerated rate of degradation, potentially shortening the duration of hypoperfusion [15]. For this reason, such HA mixtures have traditionally shown limited injury potential in the skin, though highly susceptible tissues, such as the retina, are still at significant risk for injury given their short ischemic-tolerance times [41,44,45].

In contrast, the remaining fillers tested in this study (Intense, RHA4, and Redensity) demonstrated the more brittle behavior expected of viscoelastic solids (i.e., tan(δ) < 1). The decreased pliability of these products, given their higher elastic partitions, resulted in a tendency toward small particle fragmentation both during needle/cannula extrusion and intraluminal dispersion (Figure 12B). Accordingly, the particle sizes for these fillers displayed significantly greater dependency on gel tan(δ) than any other tested physical parameter. The smallest average particle size was encountered with RHA4, which features a tan(δ) of 0.12, followed by Intense (0.39) and Redensity (0.44). Interestingly, the elastic strength (G′) of a product did not show high correlation with gel particle size, presumably because HA implants are not strong enough to resist shear stresses during cannulated extrusion into macrovascular environments. Such a feature is a necessary property of injectable HA gels, which must be able to flow during extrusion via thin needles and microcannulas [46].

**Figure 13:**
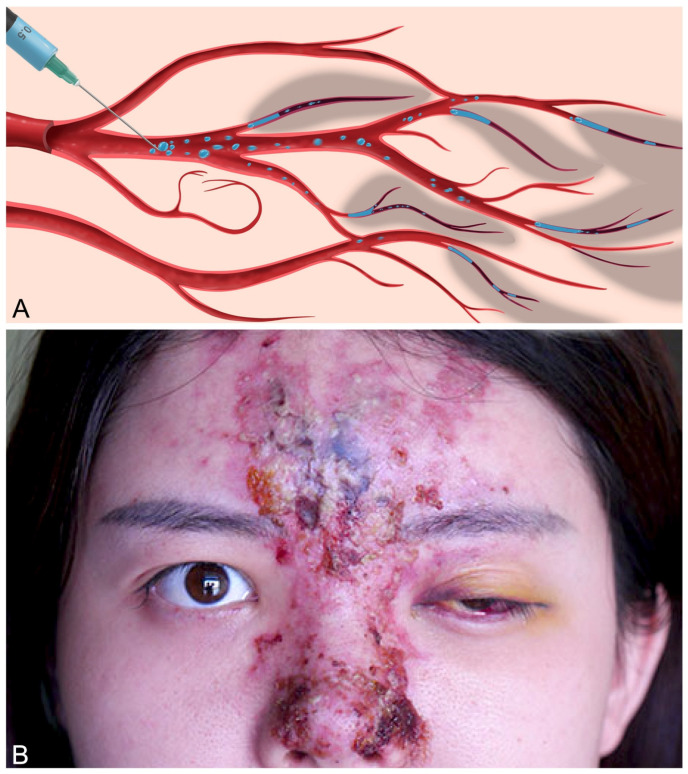
The potential effects of micro-embolic, microvascular dissemination characteristics of a gel on the ischemic injury patterns of the skin: (**A**) The dissemination of gel microparticles, and their subsequent fragmentation, creates a semi-random distribution within angiosomal microvascular networks that is influenced by the caliber of individual branches and their corresponding flow rates, as well as filler rheology, particle dimensions, and inoculation volume. Ultimately, the resulting obstructive pattern is characterized by a combination of patent and occluded proximal and distal branches, leading to a variegated distribution of skin ischemia with the percentage of patent branches decreasing with increasing inoculation volume. Adapted with permission from Soares [1] 2022, MDPI. (**B**) Appearance of skin injury in a patient 3 days following HA gel injections into the nasal dorsum showing variegated dermal discoloration characteristic of intravascular embolization, with unaffected areas present within affected territories. Reproduced with permission from Salval et al. [13] 2017, Springer Nature.

The higher elastic partition and/or strength of Intense, RHA4, and Redensity also correlated with their greater tendency toward complete branch occlusion. Although no product was able to sustain complete system obstruction at medium and high flow rates, all three products resulted in partial occlusion through blockade of a first-generation branch, preventing distal flow within the affected segment. Products with the lowest tan(δ), specifically RHA4 and Intense, resulted in relatively longer filler plugs compared to Redensity, which features a lower elastic strength and higher tan(δ). Proximal macrovascular occlusion therefore appears to be facilitated by increased gel strength and elastic partition, as well as decreased flow rates. This dynamic interplay between flow parameters and mechanical gel properties likely creates a relatively random distribution of patent and occluded daughter branches as the filler disperses distally within an angiosome, resulting in irregular patterns of dysperfusion (Figure 13). Such an irregular distribution of occlusive microparticles within an affected dermal angiosome likely contributes to the similarly irregular appearance of ischemic skin in instances of filler-induced vascular occlusion, generally referred to as *livedo racemosa* [13,47].

The findings presented in this study offer new clinical insight into the characterization of HA dermal fillers as iatrogenic embolic agents, with significant potential ramifications for our understanding of the mechanism of filler-associated ischemic injuries and relevant therapeutic interventions. Etiologically, the fragmentation of all gels into filler microparticles of varying sizes suggests that a distal, microvascular perfusion deficit plays a role in the mechanism of injury. This was further confirmed by the inability of all gel inoculations to achieve any degree of permanent macrovascular/proximal occlusion at physiologically medium and high flow rates, with branch occlusion occurring only at low flow. Resultantly, the embolic occlusive target is likely directed at vessels ranging from 100 to 1000 μm in diameter, but potentially smaller, given that a microfluidic network was not experimentally employed in this study. Certainly, the identification of microparticles <50 μm suggests a role for an additional distal microvascular injury capable of directly impacting distal arterioles and capillaries. These smaller microparticles may arise from the original inoculation or dislodge from proximally impacted gel plugs, which can act as reservoirs for secondary embolization, especially as the gel is partially degraded enzymatically and mechanically dispersed. These findings support the treatment of affected tissue beds through extravascular “flooding” of ischemic soft tissues with hyaluronidase, which have repeatedly demonstrated clinical efficacy, even if dosing standards have not yet been fully established [23,29,48]. Such targeting of the distal microvasculature maximizes surface area exposure for gel degradation of distal emboli, while limiting diffusion distances across vessel walls [49]. Nonetheless, given that proximal gel plugs are possible with large-volume inoculations of low-tan(δ) gels into low-flow vessels, simultaneous ultrasound-guided arterial mapping of proximal branches with selective treatment of occluded segments is likely to be of added therapeutic value in some instances, as suggested by several recent case series [50,51].

Several limitations of the present study exist, which relate to the complex multitude of variables that affect these types of injuries, including gel rheology, inoculation parameters, fluid mechanics of affected networks, and the functional biological nature of arterial systems. Critically, the volume and rate parameters of arterial inoculations are likely to influence the probability of proximal occlusion with any given product, variations of which were not explored in this study. For example, the rapid introduction of a large amount of gel proximally can lead to an accelerated increase in BMF viscosity, halting all flow through the affected arterial segment proximally. In this study, the rate of inoculation was kept within a “natural-feel” range of clinical injection at 0.01 mL/s, with a uniform bolus size of 0.2 mL used for all inoculations, but higher rates may be more likely to induce proximal obstruction. Similarly, the inoculation bolus size is also likely to alter the intra-arterial dissemination of fillers and their ultimate distribution within arterial networks. Additionally, given that gel properties can be partially altered by extrusion via small bore needles and microcannulas, the instrument used to inoculate an artery could shape the dynamic process of filler dispersal and micro-emboli formation [52,53]. Larger-diameter instruments may result in a predominance of larger particle sizes, facilitating proximal occlusion in physiological environments, while thinner needles may predispose to greater microfragmentation with distal embolic effects. Finally, given recent evidence suggesting a role for the coagulation system in exacerbating the occlusive potential of intra-arterial gel mixtures, the artificial design of our system, which employs a non-hematological BMF simulant, precludes the assessment of this component [28,29,30,31,54]. The elucidation of the role of these additional variables will serve as an impetus for additional studies of HA gel inoculations employing microfluidic networks, variations in needle/cannula size and length, a range of injection volumes and rates, and physiological circulating fluids.

## 4. Conclusions

This study is the first to evaluate the role of gel parameters on the dispersal behavior of HA fillers within an artificial model of arterial embolism. The use and validation of the PULSAR system allowed for precise control and simulation of physiological conditions, thus providing a clinically relevant platform for evaluating the intravascular behavior of HA fillers. The experimental results demonstrate that HA fillers undergo substantial fragmentation upon intra-arterial injection, producing ovoid embolic particles of varying sizes, with the highest frequency observed between 60–800 μm in minor axis and 90–1400 μm in major axis. Fillers with higher tan(δ) values exhibited greater microfragmentation, suggesting that gel strength and viscoelastic partitioning play an important role in determining particle size and dispersibility following intravascular injection. HA gels with lower tan(δ) values yielded smaller, more uniform fragments due to more brittle failure, whereas those with higher tan(δ) produced larger, filamentous strands due to their increased pliability. Altering flow rates within a physiological range did not significantly influence the size distribution of embolic particles, indicating that the physical properties of the gels predominantly govern their fragmentation behavior. Nonetheless, decreased flow-rate settings showed a greater predisposition toward complete, proximal branch occlusion, whereas physiologically medium-to-high facial arterial flow rates resisted proximal macrovascular occlusion altogether. Ultimately, this research advances our understanding of iatrogenic arterial embolization by HA fillers and suggests a mechanism by which micro-emboli disperse and cover larger surface areas within a functional angiosome distal to the site of cannulation, leading to cerebroretinal ischemia. The insights gained from this study could inform the development of therapeutic protocols, potentially reducing the incidence of severe complications such as tissue ischemia, blindness, and stroke. Future studies should explore the role of additional rheological, vascular, and inoculation variables, including gel cohesion, microvascular networks, and inoculation volume, rate, and needle/cannula gauge/length.

## 5. Materials and Methods

### 5.1. Vascular Model Design and Assembly

**Figure 14:**
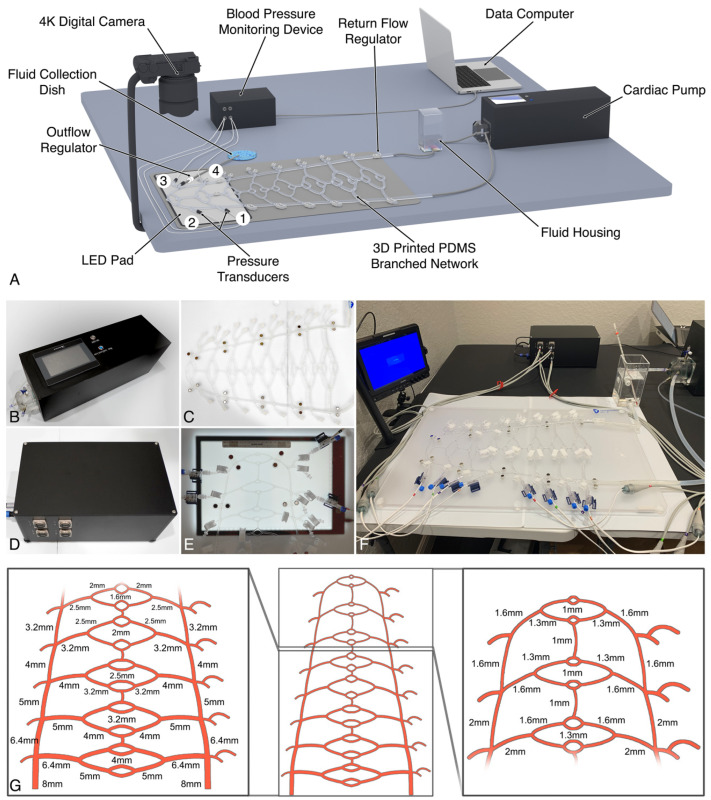
Experimental setup and components of the Pulsatile Unit for the Simulation of Arterio-embolic Restrictions (PULSAR) System: (**A**) The system is composed of a pulsatile pump attached to a 3D-printed polydimethylsiloxane dichotomously branched tubular network obeying Murray’s law of minimum work. The distal portion of the circuit is designed for simulation of the paramandibular facial artery and its proximal branches, with a vessel diameter ranging from 2 mm down to 1 mm. The circulating fluid, composed of 50% glycerol and water by volume, is stored in the fluid housing and recirculated within the system at 20 °C. Four different ports allow access to the system for inoculation (port 1), circulating fluid collection (port 3), and blood pressure monitoring (ports 1–4). The videographic capture of gel embolization employs a high-resolution digital camera tracking the movement of dyed gel particles against a lighted background. Two different regulators (return flow and outflow) allow for blood pressure and flow adjustments, which are tracked through a monitoring device connected to a receiving computer. Particle size analysis evaluated the gel-inoculated fluid directly sampled from the port 3 collection outflow. (**B**–**F**) System components showing the pulsatile pump, 3D-printed circuit, blood pressure monitoring device, distal circuit setup, and whole circuit setup, respectively. (**G**) Diagram of the tubular network showing the branch inner diameters.

An experimental physiological model of the human soft tissue macrocirculation was developed to simulate the pulsatile, laminar flow environment of the distal common carotid and proximal facial arterial branches (Pulsatile Unit for the Laboratory Simulation of Arterio-embolic Restrictions, PULSAR), as illustrated in Figure 14. The components of the system include a modified cardiac pump (EDU-Heart Pump, Trandomed, Ningbo, China), calibrated to mimic the range of flow rates observed in the human common carotid artery, connected to a 3D-printed polydimethylsiloxane (PDMS) tubular branched circuit (Peripheral Vessel Model (custom), Trandomed, Ningbo, China) [55]. The circuit, composed of Shore A-44 PDMS elastomer with a longitudinal tensile strength of 4.91 MPa, falling within the range for small to medium arteries, incorporates an initial input diameter of 8 mm followed by seven sequential branching generations obeying Murray’s law of minimum work, resulting in progressively narrower branches, down to the smallest diameter of 1 mm [56,57,58]. Each main branching generation was designed with a twice-bifurcating “vascular bed” interconnected by anastomotic branches, with the distal beds mimicking the facial artery and its proximal branches (range of ~1–2 mm in diameter). The design incorporates 24 individual access ports—8 on the supply side and 16 on the return side of the vascular beds—to facilitate fluid sampling, pressure monitoring, and diameter-specific outflow extensions. Continuous fluid-pressure measurements were recorded directly from fluid-pressure transducers connected to each respective port, processed via an 8-channel monitoring system (BP Monitoring System, Trandomed, Ningbo, China) featuring a sampling rate of 4000 Hz and a range of 0–200 mmHg.

### 5.2. System Priming with Blood-Mimicking Fluid

The circulating blood-mimicking fluid (BMF) consisted of a 50% mixture, by volume, of glycerol and water. This mixture was prepared by combining 500 mL of 99.5% glycerol with 500 mL of deionized distilled water (Sigma-Aldrich, Inc., St. Louis, MO, USA), using a magnetic hot plate stirrer (Scilogex SCI340-HS, Rocky Hill, CT, USA) [59]. The glycerol was heated to 50 °C and stirred at 250 rpm. Subsequently, the distilled water was gradually introduced into the vortex and stirred at 600 rpm for 15 min or until the mixture became homogeneous. The mixture was then allowed to cool to 20 °C before circulation within the model. Once cooled, the BMF was gradually introduced into the system by first filling the pump cavity, followed by the BMF housing, and then the tubular network, with distal clamps positioned at each port to prevent leakage. All air pockets contained within the tubular network and at each port were suctioned out of the system and subsequently replaced with BMF for complete system priming.

### 5.3. System Calibration and Validation

Calibration of the PULSAR unit was performed in triplicates across a physiological range of heart rates (specifically 60, 70, 80, 90, and 100 bpm) and relevant stroke volumes (3, 4, and 4.5 mL/beat), aiming to stay within the physiological blood pressure range (95–165/65–105 mmHg) and common carotid-artery flow rates (140–620 mL/min) for the general population [60,61,62,63,64]. Pressure calibration was achieved by adjusting the outflow regulator clamp controlling the BMF return path toward the fluid housing and by control of an additional regulator (Flow Controller Extension Set, Amsino International, Pomona, CA, USA) at the distal sampling line connected to port 3 (Figure 14A). Flow rates were directly measured from the inoculation access port (port 1) over 10 s, also performed in triplicates. Blood pressure measurements were recorded over 1 min and exported into digital spreadsheets in Microsoft Excel software version 16.83 (Microsoft Corp., Redmond, WA, USA) with calculation of average systolic and diastolic pressures, mean arterial pressure (MAP), and Reynolds number for each setting (fluid density 1120 kg/m^3^, dynamic viscosity 0.00674 Pa·s, vessel diameter 0.002 m). Additionally, laminar flow was further confirmed via direct visualization through the opened distal outflow from port 1.

**Figure 15:**
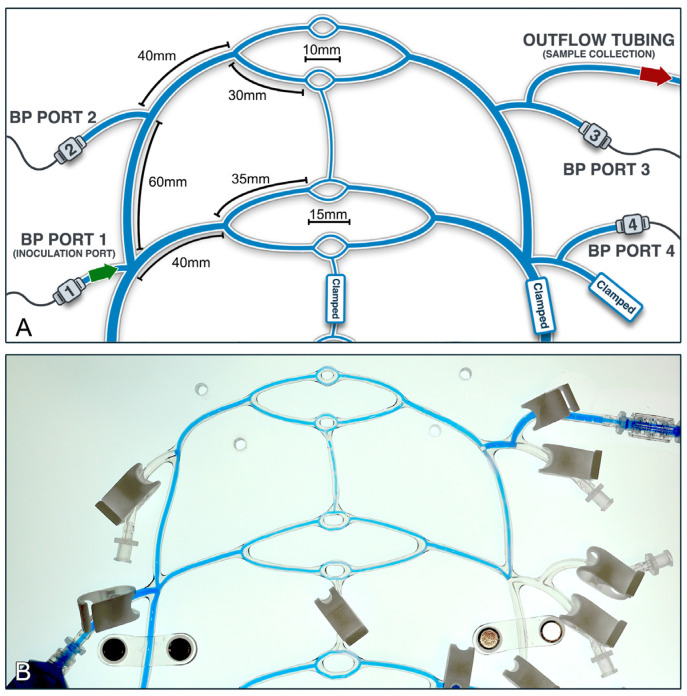
Experimental setup for simulated proximal facial artery inoculation. (**A**) Diagrammatic illustration of the distal-tubular network setup. Segments of the circuit are clamped off in order to isolate the distal circuit. Inoculation is performed through port 1 access (green arrow) and the circulating fluid containing the embolic particles is collected through port 3 (red arrow). (**B**) Photograph of the distal circuit containing dyed fluid for visualization of the experimental tubular diameters and setup, inoculation and connecting ports, and relevant clamps.

Following whole-system calibration, the circuit was further adjusted for facial artery simulation (Figure 15). Modifications included clamping the distal return tubing for the two distal vascular beds in order to isolate the distal network (encompassing the 2 mm-diameter tubing and its 1.6 mm-diameter daughter branches) for simulation of the proximal paramandibular facial artery in humans (diameter 0.9–2.5 mm) [65,66,67]. Repeat system calibration was again carried out, aiming to mimic physiological blood pressure while maintaining flow rates within the facial artery range (7.3–35.3 mL/min) [68,69,70,71]. Finally, upon completion, three different heart rate and stroke volume settings were established to span low (~10 mL/min), medium (~18 mL/min), and high (~36 mL/min) flow rates for the experimental system inoculations.

### 5.4. Gel Inoculation and Videographic Capture of Embolic Dissemination

Simulated inoculation of HA gel into the intra-arterial milieu was achieved through the delivery of a 0.2 mL bolus of four different FDA-approved dermal fillers featuring extremes of gel strength (G′) and viscoelastic partitionings (tan(δ)), chosen from two product manufacturers. Specifically, RHA Redensity and RHA4 (Revance Aesthetics, Nashville, TN, USA), representing the manufacturer’s Preserved Network Technology^®^ (PNT) crosslinking process and Belotero Revive and Belotero Intense (Merz Aesthetics, Raleigh, NC, USA), which feature Cohesive Polydensified Matrix^®^ (CPM) crosslinking technology. The chemical and physical properties of each product are listed in Table 4, with Table 5 summarizing the meaning of basic rheological parameters [72]. Table 4 data were obtained from Leffler et al.’s and Faivre et al.’s previously reported values [73,74]. Prior to inoculation, 1 mL of each gel was mixed with 100 μL of 1% aqueous toluidine blue (Carolina Biological Supply Company, Burlington, NC, USA), analogous to other aqueous dispersal protocols [75,76]. The gel product and the dye were each transferred into separate 1 mL syringes (1 mL Luer Lock Tip Syringe, Exelint, Redondo Beach, CA, USA). The gel-containing and dye-containing syringes were then connected via a Luer-to-Luer connector (Rapidfill Connector, Baxter Healthcare, Deerfield, IL, USA) and subsequently mixed back and forth 20 times until the gel was uniformly dyed.

Inoculation was performed upon activation of the PULSAR system and verification of pressure and flow parameters. The injection cannula was subsequently introduced into port 1 and the gel injected into the circulating fluid employing a 27 G, 50 mm microcannula (Steriglide, TSK Laboratories, Vancouver, BC, Canada) at a clinically appropriate extrusion rate of 0.01 mL/s, akin to other clinical/experimental evaluations [77,78]. The dispersal profile of the filler within the distal system was subsequently captured via continuous high-resolution video recording with a 4 K, 24.2 megapixel digital single-lens reflex (DSLR) camera (Alpha 6600, E18-135 mm Lens, Sony, New York, NY, USA) over a span of 5 min post-inoculation or until all of the visible inoculum dissipated from the system or resulted in terminal, static flow restriction. In between inoculations, the circulating fluid was fully cleared from the inoculated tubing and any filler remnants flushed out of the system through the fluid collection port.

### 5.5. Macroscopic and Microscopic Imaging of Embolic Gel Fragments

Macroscopic particle size evaluation was performed by collection of circulating fluid directly from the return tubing onto a 100 × 20 mm Petri dish (Pyrex Dish, Corning, Rosemont, IL, USA) equipped with a 0.01 mm glass calibrating ruler (Microscope Calibration Slide, MUHWA Scientific, Zhanjiang, China). Still images of the returning fluid were obtained for each product inoculation immediately upon collection, with a high-resolution (50 µm) photographic setup employing a 4 K, 20.9-megapixel DSLR camera (D7500, Nikon, Melville, NY, USA) and a macro lens (Nikkor 105 mm f/2.8 G, Nikon, Melville, NY, USA) backlit by an LED lighting pad (LitEnergy A4 Ultra-thin LED Lightbox, GGE Corp. Shenzhen, China). Returning fluid sampling was also microscopically evaluated for identification of microparticles <50 µm. A 100 μL sample of the returning circulating fluid containing the dispersed inoculum was evaluated via light microscopy at 100× magnification (B120 binocular compound microscope, Amscope, Irvine, CA, USA) with digital capture employing a 5-megapixel color eyepiece camera (MD-500, Amscope, Irvine, CA, USA). The particle field was scanned and randomly sampled through capture of five different view fields via dedicated microimaging software (Amlite, Amscope, Irvine, CA, USA).

### 5.6. Gel Particle Size and Clearance Analysis

**Figure 16:**
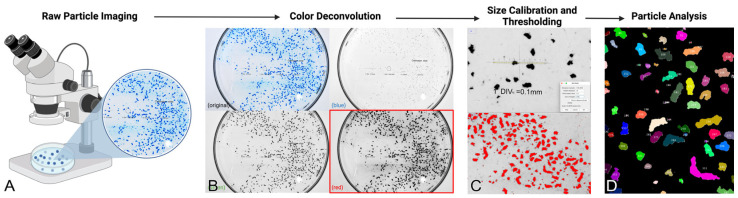
Schematic showing the protocol used to characterize HA gel fragments following inoculation into the PULSAR system: (**A**) High-resolution macroscopic and microscopic images of the HA gel fragments were taken for analysis in ImageJ. (**B**) Images underwent color-channel deconvolution. (**C**) The red channel images were calibrated and particles were thresholded. (**D**) Particle analysis was conducted on color-isolated images.

Captured high-resolution images were analyzed for particle size using ImageJ version 1.5.4 (ImageJ, U. S. National Institutes of Health, Bethesda, MD, USA). Gel remnants were quantified as the combined area encompassed by dyed fragments remaining within the system at the termination of the inoculation session, utilizing image analysis software with the following protocol. First, high-resolution macroscopic images were opened in ImageJ and underwent RGB color deconvolution to isolate the red, green, and blue image splits. The red split image was chosen for particle analysis, as it best isolates the blue particle fragments. Next, the image size was calibrated using the calibration slide glass. After calibration, a standard image threshold was applied and adjusted to threshold the in-field particle fragments. The threshold was applied, leaving white contrasted particles against a black background. Finally, using the ‘analyze particle’ tool with lower-bound 4-square-pixel particle-size cutoff, each particle’s area, major- and minor-axis lengths, perimeter, circularity, and aspect ratios were computed and overlay masks were applied to each image for visualization (Figure 16). This protocol was also used to analyze light-microscopy images. All images were opened in ImageJ and analyzed with the following workflow: Image → Color → Split Channels. On Red Channel image split: Image → Adjust → Threshold → Apply (after adjustment). Analyze → Set Measurements → Select (Area, standard deviation, integrated density, shape descriptors, perimeter). Process → Binary → Fill Holes. Process → Binary → Watershed. Define ROI with freehand selection. Analyze → Analyze Particles → Size (pixels2): 4-infinity; Select (Display results, exclude on edges, include holes, overlay, summarize) → OK.

To determine the time to clearance, high-definition videos were opened in ImageJ as audio video interleave (AVI) files. Snapshots were acquired of the AVIs at 0.1 s intervals. Each snapshot was then merged into a stack with the following command flow: Image → Stacks → Images to Stack. The generated stack had a color threshold applied to all images comprising the stack, with the following command flow: Image → Adjust → Color Threshold. The RBG and brightness thresholds were set to primarily highlight the blue dyed-gel fragments. Once the thresholds were set and applied to the stack, the following command flow was applied to quantify the time-dependent embolic burden within the observed channels: Image → Stacks → Measure Stack. Measurements were defined with the following command flow: Analyze → Set Measurements → Area, Shape Descriptor, Area Fraction, Integrated Density. The stack measurement was exported to the ImageJ results tab and plotted with the x and y axis defined as the time and % area, respectively. Thus, it was possible to quantitatively observe when the area spike related to the intravascular filler traversing the network cleared the PULSAR network. These data were graphed as columnar data.

### 5.7. Statistical Analysis

Statistical analysis was conducted using GraphPad Prism (GraphPad Prism Version 10.2.3, GraphPad Software, LLC, San Diego, CA, USA). Distribution plots for each filler were constructed without any data trimming. Either one- or two-way ANOVA analysis was used to test for significance between and within groups, when appropriate. Linear regression analysis was conducted when appropriate. Significance was denoted as * *p* < 0.05, ** *p* < 0.01, *** *p* < 0.001, and **** *p* < 0.0001. In cases where all pairwise comparisons could not fit in the graphs, the *p* values from the analyses are listed in the Supporting Information, as respectively indicated. Plotted values above each bar graph represent the mean value of that group. Data figures and illustrations were created using the BioRender design platform (BioRender, Toronto, ON, Canada).

## Figures and Tables

**Table 1:** Post-calibration PULSAR settings at physiologically relevant facial artery settings.

Parameter	Low Flow (7.4 mL/min)	Medium Flow (17.6 mL/min)	High Flow (35.8 mL/min)
**Heart Rate (beats/min)**	65	75	80
**Stroke Volume (mL/beat)**	2.8	3.5	4
**Cardiac Output (mL/min)**	182	262.5	320
**Average Systolic Pressure (mmHg)**	109.42 ± 1.04	107.96 ± 1.1	108.69 ± 1.49
**Average Diastolic Pressure (mmHg)**	91.76 ± 2.52	84.89 ± 4.39	86.2 ± 6.46
**Average Mean Arterial Pressure (mmHg)**	97.64 ± 2.52	92.58 ± 0.41	94.17 ± 1.37

**Table 2:** Summary of gel fragment dimensions and shape.

Product	Flow Setting	Area (mm^2^)	Minor Axis (µm) by Percentile				Major Axis (µm) by Percentile				Perimeter (mm)	Circularity (0–1)	Aspect Ratio
			25%	50%	75%	100%	25%	50%	75%	100%			
**Belotero** **Intense**	High	0.48 ± 0.57	149	403	808	1528	220	659	1252	3556	2.59 ± 1.28	0.69 ± 0.56	1.71 ± 0.61
	Medium	0.55 ± 0.59	216	516	751	1474	341	802	1382	3771	2.91 ± 1.49	0.67 ± 0.86	1.82 ± 0.97
	Low	0.32 ± 0.41	129	279	523	1760	197	431	832	2669	2.08 ± 0.97	0.72 ± 0.47	1.63 ± 0.52
**RHA Redensity**	High	0.14 ± 0.16	154	242	366	1034	235	375	557	1413	1.33 ± 0.86	0.78 ± 0.12	1.54 ± 0.42
	Medium	0.14 ± 0.14	187	287	421	1120	286	445	656	1490	1.37 ± 0.78	0.75 ± 0.12	1.6 ± 0.43
	Low	0.2 ± 0.18	212	383	555	1259	308	559	824	1569	1.68 ± 0.86	0.76 ± 0.11	1.59 ± 0.44
**Belotero Revive**	High	0.65 ± 1.13	79	206	436	11,354	105	345	784	17,676	2.96 ± 4.71	0.67 ± 0.80	2.06 ± 0.88
	Medium	0.77 ± 1.97	62	119	412	3867	90	145	655	14,202	3.46 ± 6.33	0.69 ± 0.24	1.98 ± 1.98
	Low	0.41 ± 0.81	63	190	501	4412	105	316	809	8573	2.31 ± 2.67	0.72 ± 0.19	1.77 ± 0.72
**RHA4**	High	0.10 ± 0.09	174	281	376	1059	271	444	606	1511	1.2 ± 0.62	0.72 ± 0.14	1.75 ± 1.37
	Medium	0.09 ± 0.10	161	248	338	845	250	389	539	1327	1.24 ± 0.70	0.65 ± 0.14	1.68 ± 0.86
	Low	0.15 ± 0.16	202	340	477	1285	290	518	717	1753	1.45 ± 0.83	0.74 ± 0.14	1.72 ± 1.62

**Table 3:** Goodness-of-fit between gel fragment size, physical properties, and flow rate.

Property	Flow Rate			Average
	High	Medium	Low	
**G′**	0.2928	0.4461	0.06997	0.2696233333
**G**″	0.01249	0.07421	0.3775	0.1547333333
**tan(δ)**	0.6641	0.9191	0.1688	0.584
**HA conc**	0.08515	0.00181	0.2689	0.11862
**Flow Rate**	-	-	-	0.0007134

**Table 4:** Physical properties of filler products employed in this study.

Filler	G′	G″	Tan(δ)	HA Concentration (mg/mL)
**Belotero Intense**	166	64.6	0.39	25.5
**RHA Redensity**	59	26	0.44	15
**Belotero Revive**	15	18.5	1.27	20
**RHA4**	263	31.6	0.12	23

**Table 5:** Rheological measurements, symbols, descriptions, and physico-clinical correlates. Adapted from McCarthy and Soares et al. [72].

Measure	Symbol	Description	Physical Clinical Correlate
Elastic (Storage) Modulus	G′	Solid-like parameter; ability to store energy through elastic deformation during shear straining.	A higher G′ indicates a stiffer gel structure that can resist permanent deformation, with a greater ability to return to its initial shape.
Viscous (Loss) Modulus	G″	Fluid-like parameter; ability to dissipate energy through viscous flow during shear straining.	A higher G′′ indicates a thicker gel that resists continuous flow, but with a greater tendency toward permanent deformation.
Phase Angle	tan(δ) (G″/G′)	Viscoelastic character of a gel based on the ratio of the viscous-to-elastic partitions (alternatively, the tangent of phase angle δ). Colloids with tan δ > 1 are more viscous than elastic, behaving more like fluids. Colloids with tan δ < 1 are more elastic than viscous, behaving more like solids.	Gels with low tan(δ) feel bouncier to the touch. In contrast, gels with a high tan(δ) feel more pliable or deformable.
Hyaluronic Acid Concentration (mg/mL)	HA Conc.	Concentration of hyaluronic acid within the gel	Influences the overall gel properties such as gel strength, viscosity, and elasticity

## Data Availability

The raw data are available from the authors upon reasonable request.

## Supplementary Materials

The following supporting information can be downloaded at: https://www.mdpi.com/article/10.3390/gels10080530/s1, Table S1: PULSAR system calibration data summary; Video S1: Demonstration of Laminar Flow from Port 1 During PULSAR System Activation; Figure S1: Microscopy of Inoculated Blood-Mimicking Fluid Revealing Microparticles Taken under bright-field microscopy; Table S2: Pairwise comparisons for each HA gel product’s fragment size at different flow rates; Table S3: 2-way ANOVA pairwise comparison between HA gel product fragment size and flow rate; Table S4: Average particle at each flow rate combining filler products; Table S5: ANOVA comparison between all fillers at combined flow rates; Table S6: ANOVA of measured perimeters; Table S7: ANOVA of measured Aspect Ratio; Table S8: ANOVA of measured circularity; Video S2: Videographic Summary of Gel Inoculations for Belotero Intense at High, Medium, and Low Flow Rates; Video S3: Videographic Summary of Gel Inoculations for RHA-4 at High, Medium, and Low Flow Rates; Video S4: Videographic Summary of Gel Inoculations for RHA Redensity at High, Medium, and Low Flow Rates; Video S5: Videographic Summary of Gel Inoculations for Belotero Revive at High, Medium, and Low Flow Rates.

## References

[1] Soares D.J. (2022). Bridging a Century-Old Problem: The Pathophysiology and Molecular Mechanisms of HA Filler-Induced Vascular Occlusion (FIVO)-Implications for Therapeutic Interventions. Molecules.

[2] Marcus F., Claude E.V., Josephine M., Teyang A. (2019). An Exceptional Cause of Acute Limb Ischemia: Nicolau Syndrome-Single-Center Experience with 4 Cases. Ann. Vasc. Surg..

[3] Yang Q., Lu B., Guo N., Li L., Wang Y., Ma X., Su Y. (2020). Fatal Cerebral Infarction and Ophthalmic Artery Occlusion After Nasal Augmentation with Hyaluronic Acid-A Case Report and Review of Literature. Aesthetic Plast. Surg..

[4] Kapoor K.M., Kapoor P., Heydenrych I., Bertossi D. (2020). Vision Loss Associated with Hyaluronic Acid Fillers: A Systematic Review of Literature. Aesthetic Plast. Surg..

[5] Kong J., Yang T., Yang X., Zhang F., Liao X., Li D. (2023). Death from Pulmonary Embolism Caused by Vaginal Injection of Hyaluronic Acid: A Case Report and a Literature Review. Aesthetic Plast. Surg..

[6] Soares D.J., Bowhay A., Blevins L.W., Patel S.M., Zuliani G.F. (2023). Patterns of Filler-Induced Facial Skin Ischemia: A Systematic Review of 243 Cases and Introduction of the Foem Scoring System and Grading Scale. Plast. Reconstr. Surg..

[7] Kim W.B., Alhusayen R.O. (2015). Skin Necrosis from Intra-articular Hyaluronic Acid Injection. J. Cutan. Med. Surg..

[8] Zhuang J., Zheng Q., Su X., Jiang L., Hu J. (2023). Clinical Manifestations and Prognosis of Embolism Caused by Filler Injection in Different Facial Regions. Plast. Reconstr. Surg. Glob. Open.

[9] Chen Y., Zhang Y.L., Luo S.K. (2019). Experimentally Induced Arterial Embolism by Hyaluronic Acid Injection: Clinicopathologic Observations and Treatment. Plast. Reconstr. Surg..

[10] Zhao F., Chen Y., He D., You X., Xu Y. (2024). Disastrous cerebral and ocular vascular complications after cosmetic facial filler injections: A retrospective case series study. Sci. Rep..

[11] Wang H.C., Yu N., Wang X., Dong R., Long X., Feng X., Li J., Wu W.T.L. (2022). Cerebral Embolism as a Result of Facial Filler Injections: A Literature Review. Aesthetic. Surg. J..

[12] Grzybinski S., Temin E. (2018). Vascular Occlusion after Hyaluronic Acid Filler Injection. Clin. Pract. Cases Emerg. Med..

[13] Salval A., Ciancio F., Margara A., Bonomi S. (2017). Impending Facial Skin Necrosis and Ocular Involvement After Dermal Filler Injection: A Case Report. Aesthetic Plast. Surg..

[14] Food and Drug Administration FDA Executive Summary General Issues Panel Meeting on Dermal Fillers. https://www.fda.gov/media/146870/download.

[15] Soares D.J., McCarthy A.D. (2024). Commentary on “Histopathologic analysis of hyaluronic acid composite solution following intravascular injection: Variability and safety”. J. Cosmet. Dermatol..

[16] de la Guardia C., Virno A., Musumeci M., Bernardin A., Silberberg M.B. (2022). Rheologic and Physicochemical Characteristics of Hyaluronic Acid Fillers: Overview and Relationship to Product Performance. Facial Plast. Surg..

[17] Wu G.T., Kam J., Bloom J.D. (2023). Hyaluronic Acid Basics and Rheology. Clin. Plast. Surg..

[18] Pluda S., Salvagnini C., Fontana A., Marchetti A., Di Lucia A., Galesso D., Guarise C. (2023). Investigation of Crosslinking Parameters and Characterization of Hyaluronic Acid Dermal Fillers: From Design to Product Performances. Gels.

[19] Faber J.E., Chilian W.M., Deindl E., van Royen N., Simons M. (2014). A brief etymology of the collateral circulation. Arter. Thromb. Vasc. Biol..

[20] Hayreh S.S. (2011). Acute retinal arterial occlusive disorders. Prog. Retin. Eye Res..

[21] Mangiardi M., Bonura A., Iaccarino G., Alessiani M., Bravi M.C., Crupi D., Pezzella F.R., Fabiano S., Pampana E., Stilo F. (2023). The Pathophysiology of Collateral Circulation in Acute Ischemic Stroke. Diagnostics.

[22] Saint-Cyr M., Wong C., Schaverien M., Mojallal A., Rohrich R.J. (2009). The perforasome theory: Vascular anatomy and clinical implications. Plast. Reconstr. Surg..

[23] DeLorenzi C. (2017). New High Dose Pulsed Hyaluronidase Protocol for Hyaluronic Acid Filler Vascular Adverse Events. Aesthet. Surg. J..

[24] Borzabadi-Farahani A., Mosahebi A., Zargaran D. (2024). A Scoping Review of Hyaluronidase Use in Managing the Complications of Aesthetic Interventions. Aesthetic Plast. Surg..

[25] Hwang C.J., Morgan P.V., Pimentel A., Sayre J.W., Goldberg R.A., Duckwiler G. (2016). Rethinking the role of nitroglycerin ointment in ischemic vascular filler complications: An animal model with ICG imaging. Ophthalmic Plast. Reconstr. Surg..

[26] Nie F., Xie H., Wang G., An Y. (2019). Risk Comparison of filler embolism between polymethyl methacrylate (PMMA) and hyaluronic acid (HA). Aesthetic Plast. Surg..

[27] Zhuang Y., Yang M., Liu C. (2016). An Islanded Rabbit Auricular Skin Flap Model of Hyaluronic Acid Injection-Induced Embolism. Aesthetic Plast. Surg..

[28] Chiang C., Zhou S., Chen C., Ho D.S., Zhang H., Liu K. (2016). Intravenous Hyaluronidase with Urokinase as Treatment for Rabbit Retinal Artery Hyaluronic Acid Embolism. Plast. Reconstr. Surg..

[29] Baley-Spindel I., Villaseñor-Villalpando E., Márquez-Espriella C., Rivera-Salgado M.I., Dávila-Díaz R. (2020). Perivascular hyaluronidase with alteplase as treatment for hyaluronic acid thrombosis. Aesthet. Surg. J..

[30] Hurkal O., Sibar S., Cenetoglu S., Tuncer S., Elmas C., Seymen C.M. (2021). Arterial Occlusion After Hyaluronic Acid Injection: Treatment with Hyaluronidase and Streptokinase. Ann. Plast. Surg..

[31] Chiang C., Zhou S., Liu K. (2016). Intravenous hyaluronidase with urokinase as treatment for arterial hyaluronic acid embolism. Plast. Reconstr. Surg..

[32] Akoglu G., Ozge G., Eşme P., Erbil H. (2020). A case report of episcleral artery embolism caused by hyaluronic acid injection into the malar area. J. Cosmet. Dermatol..

[33] Peter S., Mennel S. (2006). Retinal branch artery occlusion following injection of hyaluronic acid (Restylane). Clin. Exp. Ophthalmol..

[34] Kim Y.K., Jung C., Woo S.J., Park K.H. (2015). Cerebral Angiographic Findings of Cosmetic Facial Filler-related Ophthalmic and Retinal Artery Occlusion. J. Korean Med. Sci..

[35] Salinas-Alvarez Y., Welsh E.C., Soto-Dominguez A., Quiroga-Garza A., Hernandez-Garate Y.A.K., De-La-Garza-Castro O., Elizondo-Omaña R.E., Guzman-Lopez S. (2021). Hyaluronic Acid Embolism Treated with Subcutaneous High and Low Hyaluronidase Doses: Efficacy and Surrounding Tissue Effect. Plast. Reconstr. Surg..

[36] Borregón-Nofuentes P., Avilés-Izquierdo J.A., Martínez-Izquierdo M.Á., Ribé-Bernal L., Pulido-Pérez A., Moya-González M.D., Lázaro-Ochaita P. (2013). Livedo reticularis and skin necrosis due to hyaluronic acid embolism. JAMA Dermatol..

[37] Scott G., Khonda M., Hsu T., Rivkin A., Frank K., Fezza J., Woodward J. (2023). An Experimental Model Exhibiting Anterograde and Retrograde Vascular Occlusion of Facial Fillers to Avoid Vision Loss. Plast. Reconstr. Surg. Glob. Open..

[38] Cho K.H., Dalla Pozza E., Toth G., Bassiri Gharb B., Zins J.E. (2019). Pathophysiology Study of Filler-Induced Blindness. Aesthetic Surg. J..

[39] Ugradar S. (2021). Quantifying the Digestion of Cross-Linked Hyaluronic Acid Fillers with Hyaluronidase. Dermatol. Surg..

[40] Kablik J., Monheit G.D., Yu L., Chang G., Gershkovich J. (2009). Comparative physical properties of hyaluronic acid dermal fillers. Dermatol. Surg..

[41] Zhang Y., Chen Y., Wang S., Niu H., Yu H., Luo S. (2023). Histopathologic analysis of hyaluronic acid composite solution following intravascular injection: Variability and safety. J. Cosmet. Dermatol..

[42] Faivre J., Gallet M., Tremblais E., Trévidic P., Bourdon F. (2021). Advanced Concepts in Rheology for the Evaluation of Hyaluronic Acid-Based Soft Tissue Fillers. Dermatol. Surg..

[43] Berríos-Hernández M., Casas-Fernández L., Blanco-Rodríguez J., Suárez-Peñaranda J.M. (2021). Dermal embolization associated with peroneal mononeuropathy: An unusual complication after hyaluronic acid intra-articular injections. Int. J. Dermatol..

[44] Hayreh S.S., Zimmerman M.B., Kimura A., Sanon A. (2004). Central retinal artery occlusion.: Retinal survival time. Exp. Eye Res..

[45] Tobalem S., Schutz J.S., Chronopoulos A. (2018). Central retinal artery occlusion—Rethinking retinal survival time. BMC Ophthalmol..

[46] Zerbinati N., Capillo M.C., Sommatis S., Maccario C., Alonci G., Rauso R., Galadari H., Guida S., Mocchi R. (2022). Rheological Investigation as Tool to Assess Physicochemical Stability of a Hyaluronic Acid Dermal Filler Cross-Linked with Polyethylene Glycol Diglycidyl Ether and Containing Calcium Hydroxyapatite, Glycine and L-Proline. Gels.

[47] Soares D.J., Hynes S.D., Yi C.H., Shah-Desai S., Irving S.C. (2024). Cosmetic Filler-Induced Vascular Occlusion: A Rising Threat Presenting to Emergency Departments. Ann. Emerg. Med..

[48] Aviv U., Haik J., Weiss N., Berl A., Ofir H., Nardini G., Cleary M., Kornhaber R., Harats M. (2020). Treatment Algorithm for Hyaluronic Acid-Related Complication Based on a Systematic Review of Case Reports, Case Series, and Clinical Experience. Craniomaxillofac. Trauma. Reconstr..

[49] Wang M., Li W., Zhang Y., Tian W., Wang H. (2017). Comparison of Intra-arterial and Subcutaneous Testicular Hyaluronidase Injection Treatments and the Vascular Complications of Hyaluronic Acid Filler. Dermatol. Surg..

[50] Schelke L.W., Velthuis P.J., Decates T., Kadouch J., Alfertshofer M., Frank K., Cotofana S. (2023). Ultrasound-Guided Targeted vs Regional Flooding: A Comparative Study for Improving the Clinical Outcome in Soft Tissue Filler Vascular Adverse Event Management. Aesthetic Surg. J..

[51] Schelke L.W., Velthuis P., Kadouch J., Swift A. (2023). Early ultrasound for diagnosis and treatment of vascular adverse events with hyaluronic acid fillers. J. Am. Acad. Dermatol..

[52] Lee W., Oh W., Moon H.J., Koh I.S., Yang E.J. (2020). Soft Tissue Filler Properties Can Be Altered by a Small-Diameter Needle. Dermatol. Surg..

[53] Goldman M.P., Few J., Binauld S., Nuñez I., Hee C.K., Bernardin A. (2020). Evaluation of Physicochemical Properties Following Syringe-to-Syringe Mixing of Hyaluronic Acid Dermal Fillers. Dermatol. Surg..

[54] Khan A., Gong L., Wang Y., Chu P.N., Qi L., Zhang J., Cui H. (2024). Combination Administration of Heparin and Nitroglycerin for the Treatment of Polycaprolactone-Induced Intravascular Embolism: A Preclinical Investigation. Aesthetic Plast. Surg..

[55] Camasão D.B., Mantovani D. (2021). The mechanical characterization of blood vessels and their substitutes in the continuous quest for physiological-relevant performances. A critical review. Mater. Today Bio.

[56] Wada T., Kodaira K., Fujishiro K., Okamura T. (1991). Correlation of common carotid flow volume measured by ultrasonic quantitative flowmeter with pathological findings. Stroke.

[57] Ebrahimi A.P. (2009). Mechanical properties of normal and diseased cerebrovascular system. J. Vasc. Interv. Neurol..

[58] Sherman T.F. (1981). On connecting large vessels to small. The meaning of Murray’s law. J. Gen. Physiol..

[59] Perrira N., Shuib A.S., Phang S.W., Muda A.S. (2022). Experimental Investigation of Blood Mimicking Fluid Viscosity for Application in 3D-Printed Medical Simulator. J. Phys. Conf. Ser..

[60] Eriksen B.O., Stefansson V.T., Jenssen T.G., Mathisen U.D., Schei J., Solbu M.D., Wilsgaard T., Melsom T. (2017). Blood pressure and age-related GFR decline in the general population. BMC Nephrol..

[61] Muntner P., Hardy S.T., Fine L.J., Jaeger B.C., Wozniak G., Levitan E.B., Colantonio L.D. (2020). Trends in Blood Pressure Control Among US Adults with Hypertension, 1999–2000 to 2017–2018. JAMA.

[62] Hippisley-Cox J., Coupland C., Brindle P. (2017). Development and validation of QRISK3 risk prediction algorithms to estimate future risk of cardiovascular disease: Prospective cohort study. BMJ.

[63] Ackroyd N., Gill R., Griffiths K., Kossoff G., Appleberg M. (1986). Quantitative common carotid artery blood flow: Prediction of internal carotid artery stenosis. J. Vasc. Surg..

[64] Likittanasombut P., Reynolds P., Meads D., Tegeler C. (2006). Volume flow rate of common carotid artery measured by Doppler method and Color Velocity Imaging Quantification (CVI-Q). J. Neuroimaging.

[65] Shen W.W., Jiao C.B., Ma J.X., Xia Y.C., Cui L.G. (2023). Evaluation of facial artery course variations, diameters, and depth by Doppler ultrasonography. J. Plast. Reconstr. Aesthetic Surg..

[66] Wang D., Xiong S., Zeng N., Wu Y. (2022). Facial Arterial Variations in Asians: A Study on Computed Tomographic Angiography. Aesthetic Surg. J..

[67] Lee S.H., Ha T.J., Koh K.S., Song W.C. (2019). External and Internal Diameters of the Facial Artery Relevant to Intravascular Filler Injection. Plast. Reconstr. Surg..

[68] Rojananin S., Igarashi T., Ratanavichitrasin A., Lertakayamanee N., Ruksamanee A. (1996). Experimental study of the facial artery: Relevance to its reverse flow competence and cutaneous blood supply of the neck for clinical use as a new flap. Head Neck.

[69] Hölzle F., Hohlweg-Majert B., Kesting M.R., Mücke T., Loeffelbein D.J., Wolff K.D., Wysluch A. (2009). Reverse flow facial artery as recipient vessel for perforator flaps. Microsurgery.

[70] Schneider S., Affeld K., Kopic C., Kertzscher U. (2016). Blood pressure measurement on the cheek. Curr. Dir. Biomed. Eng..

[71] Bettoni J., Pagé G., Salsac A.V., Constans J.M., Testelin S., Devauchelle B., Balédent O., Dakpé S. (2018). Quantitative assessment of the flow distribution in the branches of the external carotid by non-injected flow MRI. Dentomaxillofa. Radiol..

[72] McCarthy A.D., Soares D.J., Chandawarkar A., El-Banna R., Hagedorn N. (2024). Dilutional rheology of Radiesse: Implications for regeneration and vascular safety. J. Cosmet. Dermatol..

[73] Leffler K., Sattler S., Corduff N., Carroll J., Muniz M., Pecora C. (2024). Blending Rheology and Clinical Performance: Product Selection in the Cohesive Polydensified Matrix Hyaluronic Acid Fillers. Data on file.

[74] Faivre J., Wu K., Gallet M., Sparrow J., Bourdon F., Gallagher C.J. (2024). Comparison of Hyaluronidase-Mediated Degradation Kinetics of Commercially Available Hyaluronic Acid Fillers In Vitro. Aesthetic Surg. J..

[75] Sundaram H., Rohrich R.J., Liew S., Sattler G., Talarico S., Trévidic P., Molliard S.G. (2015). Cohesivity of Hyaluronic Acid Fillers: Development and Clinical Implications of a Novel Assay, Pilot Validation with a Five-Point Grading Scale, and Evaluation of Six U.S. Food and Drug Administration-Approved Fillers. Plast. Reconstr. Surg..

[76] La Gatta A., Salzillo R., Catalano C., D’Agostino A., Pirozzi A.V.A., De Rosa M., Schiraldi C. (2019). Hyaluronan-based hydrogels as dermal fillers: The biophysical properties that translate into a “volumetric” effect. PLoS ONE.

[77] Borzacchiello A., Russo L., Malle B.M., Schwach-Abdellaoui K., Ambrosio L. (2015). Hyaluronic Acid Based Hydrogels for Regenerative Medicine Applications. Biomed Res. Int..

[78] Zhou W., Hou S., Deng S., Peng Y., Fu W., Zhou Y., Yang J., Peng C. (2023). The Intrinsic Relation between the Hydrogel Structure and In Vivo Performance of Hyaluronic Acid Dermal Fillers: A Comparative Study of Four Typical Dermal Fillers. Tissue Eng. Regen. Med..

