# Supplementary material for: The Impact of Gel Parameters on the Dispersal and Fragmentation of Hyaluronic Acid Gel Fillers within an Artificial Model of Arterial Embolism
Source: Gels. 2024 Aug 12;10(8):530. doi: 10.3390/gels10080530 (PMC11353545; doi:10.3390/gels10080530)
Supplement: Supplementary file 1 [file gels-10-00530-s001.zip › Table S1.pdf]

Supplementary Table [Additional File 2] - PULSAR system calibration data summary

Heart Rate (bpm)	60				70				80			90			100	
**Stroke Volume (ml)**	3	3.5	4	4.5	3	3.5	4	4.5	3	3.5	4	3	3.5	4	3	3.5
**Pump Output (ml/min)**	180	210	240	270	210	245	280	315	240	280	320	270	315	360	300	350
**Port 2 Flow Rate (ml/min)**	11.8	16.8	31.2	41.6	9.4	16.4	31.3	44	7.6	20.4	37.4	10.1	26.4	43.2	15.1	34.6
**Reynolds Number**	20.84	29.66	54.95	73.36	16.61	28.95	55.12	77.67	13.4	35.97	65.9	17.84	46.59	76.14	26.65	60.97
	CHANNEL 1 (INOCULATION PORT)															
**Systolic Fluid Pressure (mmHg)**	111 ± 1.1	114 ± 2.6	113 ± 2.4	113 ± 3	114 ± 2.4	109 ± 0.7	108 ± 2.2	111 ± 1.2	108 ± 0.4	112 ± 0.5	111 ± 1.2	112 ± 1.2	113 ± 0.4	111 ± 1	111 ± 1	109 ± 0.1
**Diastolic Fluid Pressure (mmHg)**	88 ± 1.3	87 ± 0.3	83 ± 0.2	79 ± 0.7	92 ± 1.8	86 ± 1.6	82 ± 0.1	82 ± 4.1	91 ± 5.2	91 ± 3.1	85 ± 0.1	94 ± 0.2	96 ± 8.9	87 ± 0.1	93 ± 0.2	88 ± 1.5
**Mean Arterial Pressure (mmHg)**	95.5	96.4	92.9	90.2	99.2	93.9	90.9	91.3	96.8	98.2	93.9	100.4	101.8	94.9	99	95.4
	CHANNEL 2															
**Systolic Fluid Pressure (mmHg)**	110 ± 1.2	113 ± 1.6	111 ± 1.2	109 ± 1.9	113 ± 2.7	108 ± 0.6	106 ± 2	107 ± 1.1	108 ± 0.4	111 ± 0.5	108 ± 1.1	112 ± 1.1	111 ± 0.5	108 ± 1	110 ± 1	107 ± 0.1
**Diastolic Fluid Pressure (mmHg)**	87 ± 0.4	89 ± 5.6	81 ± 0.2	76 ± 0.6	91 ± 1.8	86 ± 1.3	81 ± 0.1	79 ± 1.2	90 ± 2.5	90 ± 3	83 ± 0.1	95 ± 0.2	91 ± 3.2	84 ± 0.1	93 ± 0.2	86 ± 0.1
**Mean Arterial Pressure (mmHg)**	94.5	96.8	90.7	87	98.2	93.4	89.1	88.1	96.3	97.4	91.7	100.7	97.9	91.8	98.4	93.4
	CHANNEL 3 (OUTFLOW PORT)															
**Systolic Fluid Pressure (mmHg)**	108 ± 0.8	109 ± 1.6	102 ± 1.3	97 ± 1.8	112 ± 2.4	104 ± 0.7	98 ± 2.1	95 ± 1.2	0.5	107 ± 0.8	99 ± 1.2	111 ± 1.2	105 ± 0.3	97 ± 1.1	107 ± 0.7	99 ± 1.1
**Diastolic Fluid Pressure (mmHg)**	84 ± 0.3	92 ± 12.8	73 ± 0.2	66 ± 0.6	89 ± 1.8	81 ± 1	72 ± 0.1	68 ± 2.7	88 ± 0.8	87 ± 6.7	74 ± 0.1	92 ± 0.2	94 ± 10.8	73 ± 0.1	89 ± 0.2	78 ± 0.6
**Mean Arterial Pressure (mmHg)**	91.8	97.5	82.7	76.1	96.3	89	81	77.2	94.2	93.8	82.3	98	98.1	81.1	95	85.2
	CHANNEL 4															
**Systolic Fluid Pressure (mmHg)**	111 ± 0.8	113 ± 1.5	112 ± 1.2	110 ± 2	114 ± 2.5	109 ± 0.8	107 ± 2.3	108 ± 1.3	109 ± 0.5	112 ± 0.8	109 ± 1.3	113 ± 1.2	112 ± 0.4	108 ± 2.3	110 ± 2.5	105 ± 3.4
**Diastolic Fluid Pressure (mmHg)**	86 ± 0.4	100 ± 13.8	89 ± 13.9	76 ± 3	91 ± 1.8	85 ± 0.1	80 ± 0.1	78 ± 0.1	89 ± 0.1	89 ± 1	83 ± 0.1	95 ± 5.4	90 ± 1.1	86 ± 6	95 ± 5.3	94 ± 8.1
**Mean Arterial Pressure (mmHg)**	94.5	104.2	96.7	87.4	98.8	93.1	89	88.1	95.8	96.9	91.6	101.3	97.3	93.7	100	97.7
	4-CHANNEL AVERAGES															
**Systolic Fluid Pressure (mmHg)**	110 ± 0.98	112 ± 1.83	109 ± 1.51	107 ± 2.15	113 ± 2.5	107 ± 0.71	105 ± 2.13	105 ± 1.2	108 ± 0.44	111 ± 0.63	107 ± 1.17	112 ± 1.16	110 ± 0.39	106 ± 1.36	110 ± 1.3	105 ± 1.18
**Diastolic Fluid Pressure (mmHg)**	86 ± 0.58	92 ± 8.1	81 ± 3.61	74 ± 1.22	91 ± 1.81	85 ± 1.0	79 ± 0.06	77 ± 2.03	90 ± 2.15	90 ± 3.44	81 ± 0.11	94 ± 1.47	93 ± 5.98	82 ± 1.58	92 ± 1.44	87 ± 2.57
**Mean Arterial Pressure (mmHg)**	94.1	98.7	90.8	85.2	98.1	92.3	87.5	86.2	95.8	96.6	89.9	100.1	98.8	90.4	98.1	92.9
**Pulse Pressure Diff (mmHg)**	23	20	28	33	23	23	26	29	18	21	25	18	17	24	17	18
