# Supplementary material for: The Impact of Gel Parameters on the Dispersal and Fragmentation of Hyaluronic Acid Gel Fillers within an Artificial Model of Arterial Embolism
Source: Gels. 2024 Aug 12;10(8):530. doi: 10.3390/gels10080530 (PMC11353545; doi:10.3390/gels10080530)
Supplement: Supplementary file 1 [file gels-10-00530-s001.zip › Tables S2-S5.pdf]

Tables S2-S5 [Additional File 4]

Table S2. Pairwise comparisons for each HA gel product's fragment size at different flow rates.

RHA4 ANOVA					
Tukey's multiple comparisons test	Mean Diff.	95.00% CI of diff.	Below threshold?	Summary	Adjusted P Value
High vs. Medium	0.002809	-0.003472 to 0.009089	No	ns	0.5464
High vs. Low	-0.05251	-0.06059 to -0.04443	Yes	****	<0.0001
Medium vs. Low	-0.05532	-0.06289 to -0.04774	Yes	****	<0.0001

Revive					
Tukey's multiple comparisons test	Mean Diff.	95.00% CI of diff.	Below threshold?	Summary	Adjusted P Value
Low vs. Medium	1.075	-2.913 to 5.062	No	ns	0.7989
Low vs. High	1.07	-2.918 to 5.057	No	ns	0.8007
Medium vs. High	-0.005215	-3.540 to 3.529	No	ns	>0.9999

Redensity					
Tukey's multiple comparisons test	Mean Diff.	95.00% CI of diff.	Below threshold?	Summary	Adjusted P Value
Low vs. Medium	0.06745	0.05275 to 0.08214	Yes	****	<0.0001
Low vs. High	0.06566	0.05153 to 0.07980	Yes	****	<0.0001
Medium vs. High	-0.001784	-0.01159 to 0.008025	No	ns	0.9047

Intense					
Tukey's multiple comparisons test	Mean Diff.	95.00% CI of diff.	Below threshold?	Summary	Adjusted P Value
Low vs. Medium	-0.2296	-0.3034 to -0.1558	Yes	****	<0.0001
Low vs. High	-0.1666	-0.2374 to -0.09581	Yes	****	<0.0001
Medium vs. High	0.06299	0.003308 to 0.1227	Yes	*	0.0357

Table S3. 2-way ANOVA pairwise comparison between HA gel product fragment size and flow rate.

Tukey's multiple comparisons test	Predicted (LS) mean diff.	95.00% CI of diff.	Below threshold?	Summary	Adjusted P Value
35.8					
Intense vs. Redensity	0.3437	0.2613 to 0.4261	Yes	****	<0.0001
Intense vs. Revive	-0.1637	-0.2881 to -0.03933	Yes	**	0.004
Intense vs. RHA4	0.386	0.3007 to 0.4714	Yes	****	<0.0001
Redensity vs. Revive	-0.5074	-0.6164 to -0.3983	Yes	****	<0.0001
Redensity vs. RHA4	0.04234	-0.01851 to 0.1032	No	ns	0.2792
Revive vs. RHA4	0.5497	0.4385 to 0.6610	Yes	****	<0.0001
17.8					
Intense vs. Redensity	0.1788	0.08471 to 0.2730	Yes	****	<0.0001
Intense vs. Revive	-0.4566	-0.6024 to -0.3109	Yes	****	<0.0001
Intense vs. RHA4	0.2222	0.1325 to 0.3120	Yes	****	<0.0001
Redensity vs. Revive	-0.6355	-0.7655 to -0.5054	Yes	****	<0.0001
Redensity vs. RHA4	0.04337	-0.01762 to 0.1044	No	ns	0.2605
Revive vs. RHA4	0.6789	0.5519 to 0.8058	Yes	****	<0.0001
7.4					
Intense vs. Redensity	0.341	0.2076 to 0.4745	Yes	****	<0.0001
Intense vs. Revive	0.1383	-0.009824 to 0.2863	No	ns	0.0773
Intense vs. RHA4	0.395	0.2723 to 0.5178	Yes	****	<0.0001

Redensity vs. Revive	-0.2027	-0.3330 to -0.07251	Yes	***	0.0004
Redensity vs. RHA4	0.05401	-0.04650 to 0.1545	No	ns	0.5115
Revive vs. RHA4	0.2568	0.1375 to 0.3760	Yes	****	<0.0001
Intense					
35.8 vs. 17.8	0.1666	0.06758 to 0.2657	Yes	***	0.0002
35.8 vs. 7.4	-0.06299	-0.1804 to 0.05445	No	ns	0.4196
17.8 vs. 7.4	-0.2296	-0.3520 to -0.1073	Yes	****	<0.0001
Redensity					
35.8 vs. 17.8	0.001784	-0.05497 to 0.05854	No	ns	0.997
35.8 vs. 7.4	-0.06566	-0.1474 to 0.01609	No	ns	0.1437
17.8 vs. 7.4	-0.06745	-0.1525 to 0.01757	No	ns	0.1507
Revive					
35.8 vs. 17.8	-0.1263	-0.2704 to 0.01775	No	ns	0.0994
35.8 vs. 7.4	0.239	0.1073 to 0.3706	Yes	****	<0.0001
17.8 vs. 7.4	0.3653	0.2205 to 0.5101	Yes	****	<0.0001
RHA4					
35.8 vs. 17.8	0.002809	-0.05157 to 0.05718	No	ns	0.992
35.8 vs. 7.4	-0.05399	-0.1233 to 0.01534	No	ns	0.1613
17.8 vs. 7.4	-0.0568	-0.1222 to 0.008579	No	ns	0.1036

Table S4. Average particle at each flow rate combining filler products.

Average particle size by flow					
Tukey's multiple comparisons test	Mean Diff.	95.00% CI of diff.	Below threshold?	Summary	Adjusted P Value
35.8 vs. 17.8	0.01122	-0.4999 to 0.5224	No	ns	0.9979
35.8 vs. 7.4	0.01408	-0.4971 to 0.5252	No	ns	0.9967
17.8 vs. 7.4	0.002858	-0.5083 to 0.5140	No	ns	0.9999

Table S5. ANOVA comparison between all fillers at combined flow rates.

Tukey's multiple comparisons test	Mean Diff.	95.00% CI of diff.	Below threshold?	Summary	Adjusted P Value
Intense vs. Redensity	0.2878	0.2833 to 0.2924	Yes	****	<0.0001
Intense vs. Revive	-0.1607	-0.1670 to -0.1544	Yes	****	<0.0001
Intense vs. RHA4	0.3344	0.3300 to 0.3389	Yes	****	<0.0001
Redensity vs. Revive	-0.4485	-0.4541 to -0.4430	Yes	****	<0.0001
Redensity vs. RHA4	0.04658	0.04343 to 0.04972	Yes	****	<0.0001
Revive vs. RHA4	0.4951	0.4897 to 0.5006	Yes	****	<0.0001
