# Supplementary material for: The Impact of Gel Parameters on the Dispersal and Fragmentation of Hyaluronic Acid Gel Fillers within an Artificial Model of Arterial Embolism
Source: Gels. 2024 Aug 12;10(8):530. doi: 10.3390/gels10080530 (PMC11353545; doi:10.3390/gels10080530)
Supplement: Supplementary file 1 [file gels-10-00530-s001.zip › Tables S6-S8.pdf]

Tables S6-S8 [Additional File 5]

Table S6. ANOVA of measured perimeters.

Perimeter					
Tukey's multiple comparisons test	Predicted (LS) mean diff.	95.00% CI of diff.	Below threshold?	Summary	Adjusted P Value
Low					
Intense vs. Redensity	0.3965	0.1728 to 0.6202	Yes	****	<0.0001
Intense vs. Revive	-0.2278	-0.4745 to 0.01898	No	ns	0.0826
Intense vs. RHA4	0.6249	0.4302 to 0.8195	Yes	****	<0.0001
Redensity vs. Revive	-0.6243	-0.8409 to -0.4076	Yes	****	<0.0001
Redensity vs. RHA4	0.2283	0.07365 to 0.3830	Yes	***	0.0009
Revive vs. RHA4	0.8526	0.6661 to 1.039	Yes	****	<0.0001
Medium					
Intense vs. Redensity	1.541	1.383 to 1.698	Yes	****	<0.0001
Intense vs. Revive	-0.5468	-0.7911 to -0.3024	Yes	****	<0.0001
Intense vs. RHA4	1.678	1.527 to 1.828	Yes	****	<0.0001
Redensity vs. Revive	-2.087	-2.305 to -1.869	Yes	****	<0.0001
Redensity vs. RHA4	0.1369	0.03465 to 0.2391	Yes	**	0.0033
Revive vs. RHA4	2.224	2.012 to 2.437	Yes	****	<0.0001
High					

Intense vs. Redensity	1.262	1.123 to 1.400	Yes	****	<0.0001
Intense vs. Revive	-0.3738	-0.5840 to -0.1636	Yes	****	<0.0001
Intense vs. RHA4	1.387	1.230 to 1.545	Yes	****	<0.0001
Redensity vs. Revive	-1.635	-1.820 to -1.451	Yes	****	<0.0001
Redensity vs. RHA4	0.1258	0.003888 to 0.2476	Yes	*	0.0401
Revive vs. RHA4	1.761	1.561 to 1.961	Yes	****	<0.0001
Intense					
Low vs. Medium	-0.8355	-1.041 to -0.6304	Yes	****	<0.0001
Low vs. High	-0.5104	-0.7073 to -0.3136	Yes	****	<0.0001
Medium vs. High	0.3251	0.1591 to 0.4911	Yes	****	<0.0001
Redensity					
Low vs. Medium	0.3087	0.1662 to 0.4512	Yes	****	<0.0001
Low vs. High	0.3547	0.2177 to 0.4917	Yes	****	<0.0001
Medium vs. High	0.04602	-0.04911 to 0.1411	No	ns	0.4932
Revive					
Low vs. Medium	-1.155	-1.396 to -0.9130	Yes	****	<0.0001
Low vs. High	-0.6564	-0.8771 to -0.4358	Yes	****	<0.0001
Medium vs. High	0.4981	0.2554 to 0.7408	Yes	****	<0.0001
RHA4					

Low vs. Medium	0.2172	0.1260 to 0.3083	Yes	****	<0.0001
Low vs. High	0.2521	0.1359 to 0.3683	Yes	****	<0.0001
Medium vs. High	0.03491	-0.07468 to 0.1445	No	ns	0.7356

Table S7. ANOVA of measured Aspect Ratio.

Aspect Ratio					
Tukey's multiple comparisons test	Predicted (LS) mean diff.	95.00% CI of diff.	Below threshold?	Summary	Adjusted P Value
Low					
Intense vs. Redensity	0.03581	-0.1049 to 0.1766	No	ns	0.9143
Intense vs. Revive	-0.1433	-0.2986 to 0.01198	No	ns	0.0827
Intense vs. RHA4	-0.09043	-0.2129 to 0.03204	No	ns	0.2293
Redensity vs. Revive	-0.1791	-0.3154 to -0.04277	Yes	**	0.0041
Redensity vs. RHA4	-0.1262	-0.2236 to -0.02889	Yes	**	0.0048
Revive vs. RHA4	0.05286	-0.06450 to 0.1702	No	ns	0.6539
Medium					
Intense vs. Redensity	0.2148	0.1156 to 0.3141	Yes	****	<0.0001
Intense vs. Revive	-0.1652	-0.3189 to -0.01145	Yes	*	0.0295
Intense vs. RHA4	0.1349	0.04022 to 0.2295	Yes	**	0.0014
Redensity vs. Revive	-0.38	-0.5172 to -0.2429	Yes	****	<0.0001

Redensity vs. RHA4	-0.07997	-0.1443 to -0.01565	Yes	**	0.0077
Revive vs. RHA4	0.3001	0.1662 to 0.4339	Yes	****	<0.0001
High					
Intense vs. Redensity	0.1686	0.08168 to 0.2555	Yes	****	<0.0001
Intense vs. Revive	-0.3457	-0.4779 to -0.2134	Yes	****	<0.0001
Intense vs. RHA4	-0.03892	-0.1382 to 0.06039	No	ns	0.7453
Redensity vs. Revive	-0.5143	-0.6305 to -0.3980	Yes	****	<0.0001
Redensity vs. RHA4	-0.2075	-0.2842 to -0.1308	Yes	****	<0.0001
Revive vs. RHA4	0.3068	0.1810 to 0.4325	Yes	****	<0.0001
Intense					
Low vs. Medium	-0.1861	-0.3152 to -0.05708	Yes	**	0.0021
Low vs. High	-0.08201	-0.2059 to 0.04186	No	ns	0.2669
Medium vs. High	0.1041	-0.0003339 to 0.2086	No	ns	0.051
Redensity					
Low vs. Medium	-0.007088	-0.09676 to 0.08258	No	ns	0.9813
Low vs. High	0.0508	-0.03542 to 0.1370	No	ns	0.351
Medium vs. High	0.05788	-0.001974 to 0.1177	No	ns	0.0606
Revive					
Low vs. Medium	-0.208	-0.3600 to -0.05609	Yes	**	0.0038

Low vs. High	-0.2844	-0.4233 to -0.1455	Yes	****	<0.0001
Medium vs. High	-0.07637	-0.2291 to 0.07634	No	ns	0.4699
RHA4					
Low vs. Medium	0.03918	-0.01817 to 0.09653	No	ns	0.2451
Low vs. High	-0.0305	-0.1036 to 0.04262	No	ns	0.591
Medium vs. High	-0.06968	-0.1386 to -0.0007226	Yes	*	0.047

Table S8. ANOVA of measured circularity.

Circularity					
Tukey's multiple comparisons test	Predicted (LS) mean diff.	95.00% CI of diff.	Below threshold?	Summary	Adjusted P Value
Low					
Intense vs. Redensity	-0.03804	-0.08216 to 0.006091	No	ns	0.1193
Intense vs. Revive	-0.002948	-0.05163 to 0.04573	No	ns	0.9987
Intense vs. RHA4	-0.01478	-0.05318 to 0.02361	No	ns	0.7557
Redensity vs. Revive	0.03509	-0.007652 to 0.07783	No	ns	0.1501
Redensity vs. RHA4	0.02325	-0.007263 to 0.05377	No	ns	0.2042
Revive vs. RHA4	-0.01183	-0.04863 to 0.02496	No	ns	0.842
Medium					

Intense vs. Redensity	-0.08348	-0.1146 to -0.05236	Yes	****	<0.0001
Intense vs. Revive	-0.01899	-0.06718 to 0.02921	No	ns	0.7424
Intense vs. RHA4	0.01683	-0.01285 to 0.04650	No	ns	0.4639
Redensity vs. Revive	0.0645	0.02150 to 0.1075	Yes	***	0.0007
Redensity vs. RHA4	0.1003	0.08015 to 0.1205	Yes	****	<0.0001
Revive vs. RHA4	0.03581	-0.006151 to 0.07778	No	ns	0.1253
High					
Intense vs. Redensity	-0.08741	-0.1147 to -0.06015	Yes	****	<0.0001
Intense vs. Revive	0.01535	-0.02611 to 0.05682	No	ns	0.7771
Intense vs. RHA4	-0.03424	-0.06538 to -0.003109	Yes	*	0.0244
Redensity vs. Revive	0.1028	0.06632 to 0.1392	Yes	****	<0.0001
Redensity vs. RHA4	0.05317	0.02912 to 0.07721	Yes	****	<0.0001
Revive vs. RHA4	-0.04959	-0.08902 to -0.01016	Yes	**	0.0068
Intense					
Low vs. Medium	0.05361	0.01316 to 0.09407	Yes	**	0.0054
Low vs. High	0.03336	-0.005473 to 0.07219	No	ns	0.1089
Medium vs. High	-0.02025	-0.05300 to 0.01249	No	ns	0.3155
Redensity					
Low vs. Medium	0.008165	-0.01995 to 0.03628	No	ns	0.7747

Low vs. High	-0.01601	-0.04304 to 0.01102	No	ns	0.347
Medium vs. High	-0.02418	-0.04294 to -0.005411	Yes	**	0.0071
Revive					
Low vs. Medium	0.03758	-0.01006 to 0.08521	No	ns	0.1538
Low vs. High	0.05166	0.008130 to 0.09519	Yes	*	0.015
Medium vs. High	0.01408	-0.03379 to 0.06196	No	ns	0.7697
RHA4					
Low vs. Medium	0.08522	0.06724 to 0.1032	Yes	****	<0.0001
Low vs. High	0.0139	-0.009025 to 0.03682	No	ns	0.3299
Medium vs. High	-0.07132	-0.09294 to -0.04971	Yes	****	<0.0001
