# Supplementary figures and images for: The Impact of Gel Parameters on the Dispersal and Fragmentation of Hyaluronic Acid Gel Fillers within an Artificial Model of Arterial Embolism
Source: Gels. 2024 Aug 12;10(8):530. doi: 10.3390/gels10080530 (PMC11353545; doi:10.3390/gels10080530)

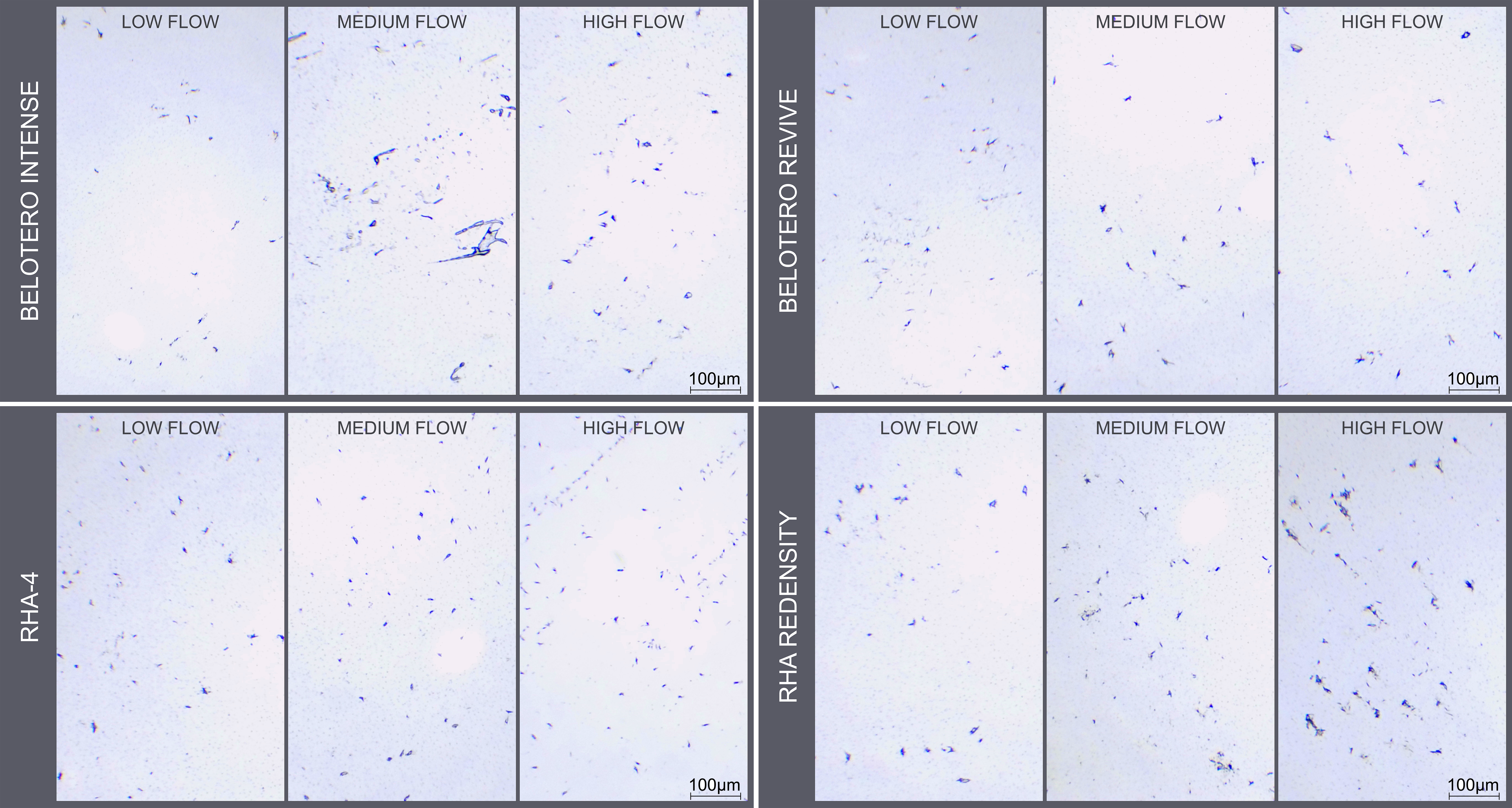

Supplement: Supplementary file 1 [file gels-10-00530-s001.zip › Figure S1.tif]
